# Pro-inflammatory cytokines can act as intracellular modulators of commensal bacterial virulence

**DOI:** 10.1098/rsob.130048

**Published:** 2013-10

**Authors:** Jafar Mahdavi, Pierre-Joseph Royer, Hong S. Sjölinder, Sheyda Azimi, Tim Self, Jeroen Stoof, Lee M. Wheldon, Kristoffer Brännström, Raymond Wilson, Joanna Moreton, James W. B. Moir, Carina Sihlbom, Thomas Borén, Ann-Beth Jonsson, Panos Soultanas, Dlawer A. A. Ala'Aldeen

**Affiliations:** 1 School of Life Sciences, Molecular Bacteriology and Immunology Group, University of Nottingham, Nottingham NG7 2RD, UK; 2 Department of Genetic, Microbiology and Toxicology (GMT), Stockholm University, 109 61 Stockholm, Sweden; 3 School of Life Sciences, Institute of Cell Signalling, University of Nottingham, Nottingham NG7 2UH, UK; 4 Department of Medical Biochemistry and Biophysics, Umeå University, 901 87 Umeå, Sweden; 5 Deep Seq, Centre for Genetics and Genomics, University of Nottingham, Queen's Medical Centre, Nottingham NG7 2UH, UK; 6 Department of Biology, University of York, Heslington, York YO10 5YW, UK; 7 Proteomics Core Facility, Sahlgrenska Academy, University of Gothenburg, PO Box 413, 405 30 Gothenburg, Sweden; 8 School of Chemistry, Centre for Biomolecular Sciences, University of Nottingham, Nottingham NG7 2RD, UK

**Keywords:** *Neisseria meningitidis*, type IV pili, glycosylation, cytokines, patho-adaptation, transcriptional regulators

## Abstract

Interactions between commensal pathogens and hosts are critical for disease development but the underlying mechanisms for switching between the commensal and virulent states are unknown. We show that the human pathogen *Neisseria meningitidis*, the leading cause of pyogenic meningitis, can modulate gene expression via uptake of host pro-inflammatory cytokines leading to increased virulence. This uptake is mediated by type IV pili (Tfp) and reliant on the PilT ATPase activity. Two Tfp subunits, PilE and PilQ, are identified as the ligands for TNF-α and IL-8 in a glycan-dependent manner, and their deletion results in decreased virulence and increased survival in a mouse model. We propose a novel mechanism by which pathogens use the twitching motility mode of the Tfp machinery for sensing and importing host elicitors, aligning with the inflamed environment and switching to the virulent state.

## 2. Introduction

*Neisseria meningitidis* (*Nm*), a Gram-negative diplococcus, is an obligate human pathogen that colonizes the nasopharyngeal epithelium and carried asymptomatically in the nasopharynx of up to 30% of the global population at a given time [1]. It is the leading cause of pyogenic meningitis, epidemic meningitis and sepsis worldwide [2,3]. The interplay between host pro-inflammatory responses and bacteria probably modulates disease in commensal and opportunistic infections, including *Nm* [4]. Recently, Wu *et al*. [5] demonstrated that IFN-γ binds to a *Pseudomonas aeruginosa* outer-membrane protein, OprF, resulting in the expression of a quorum-sensing-dependent virulence determinant, the PA-I lectin. An early investigation by Porat *et al*. [6] demonstrated that the cell–surface interaction between IL-1β and putative IL-1β receptors on virulent strains of *E. coli* enhances proliferation of these strains. More recently, Lee *et al*. [7] provided evidence for TNF-α-dependent increased growth of *E. coli* both *in vitro* and *in vivo*, but no underlying mechanism was proposed.

*Nm* expresses virulence factors that allow pathogenic strains to traverse the nasopharyngeal mucosa, disseminate into the bloodstream, and then cross the blood–brain barrier. These virulence factors are broadly grouped into two families: the major adhesin group (which includes Tfp [8]) and the minor adhesin group [2,9].

The meningococcal Tfp is a multi-functional organelle consisting of up to 18 different protein subunits [10]. The PilE (pilin) and PilQ subunits are homopolymers of the major subunit. During pilus assembly, polymerized PilE fibres are transported to the bacterial surface through the membrane-embedded dodecameric PilQ pores [11]. Meningococcal and gonococci pilins can be glycosylated, with an *O*-linked trisaccharide between amino acid residues 45 and 73 of meningococcal pilin [12]. This structure contains a terminal 1,4-linked digalactose covalently linked to a 2,4-diacetamido-2,4,5-trideoxyhexose. Type IV pili (Tfp) are involved in the initial adhesion of bacteria to host tissues during colonization [13], but pilin glycosylation does not play a major role in piliation [14]; however, it is important for pilus-mediated adhesion to human bronchial epithelial cells and tissues [15]. PilQ, on the other hand, mediates bacterial binding to laminin receptors of the host cells [16] and forms a conduit for pilus retraction [17]. A phenomenon termed ‘twitching motility’ is manifested as intermittent darting translocation of cells in an ATPase-dependent manner using the protein PilT [18]. These properties are crucial for microcolony formation, natural transformation, physical pulling of bacteria onto host cell membranes and/or invasion [19]. Proliferation of *Nm* in contact with host cells increases the transcription of the *pptB* gene encoding a transferase that adds phosphoglycerol onto Tfp. This post-translational modification is a Tfp-dependent contact between bacteria which use this regulated detachment process to propagate to new colonization sites and migrate across the epithelium during invasion [20].

An important factor that may potentially alter commensal behaviour towards pathogenicity is the inflammatory state of the host [13]. Moreover, environmental factors such as syncytial virus infection can influence inflammatory responses, and thus trigger meningitis development [21]. The pro-inflammatory response to different subtypes and serotypes of *Nm* is variable. Sialic acid incorporation into *l*ipo*o*ligo*s*accharides (LOS) [22] and capsule polysaccharide structures endows the bacteria with resistance to complement and phagocytic killing. Several studies have shown a close relationship between severity and fatality of meningococcal sepsis with plasma levels of LOS [23] and inflammation markers such as interleukin (IL)-1, IL-6, IL-8 and TNF-α [24,25]. In particular, TNF-α is associated with many of the manifestations of meningococcemia.

*Nm* is highly adaptable to its environment; it downregulates pili expression and capsule production after contact with host cells [26]. Transcriptome analysis of *Nm* in contact with epithelial and endothelial cells has revealed altered expression levels of certain genes involved in pathogenesis, such as IgA_1_ protease, proteins involved in iron uptake and Tfp assembly [27]. The coordination of capsule formation and pili gene expression maximizes the virulence and transmission of *Nm*.

The effects of *Nm* on the host and the inflammatory response caused by *Nm* infection have been investigated at length; what remains to be considered is how these cytokine-orchestrated immune responses influence *Nm* and modulate virulence gene expression and phenotype. Here, we investigate the bacterial mechanisms employed to respond to this inflammatory reaction and phenotypic change independently of any cell contact. We show that detection of environmental changes and recognition by *Nm* ligands, followed by uptake of cytokines, are key steps in the conversion of commensal bacteria into hypervirulent phenotypes.

## 3. Results

### 3.1. *Neisseria meningitidis* type IV pili bind to human cytokines

**Figure 1:**
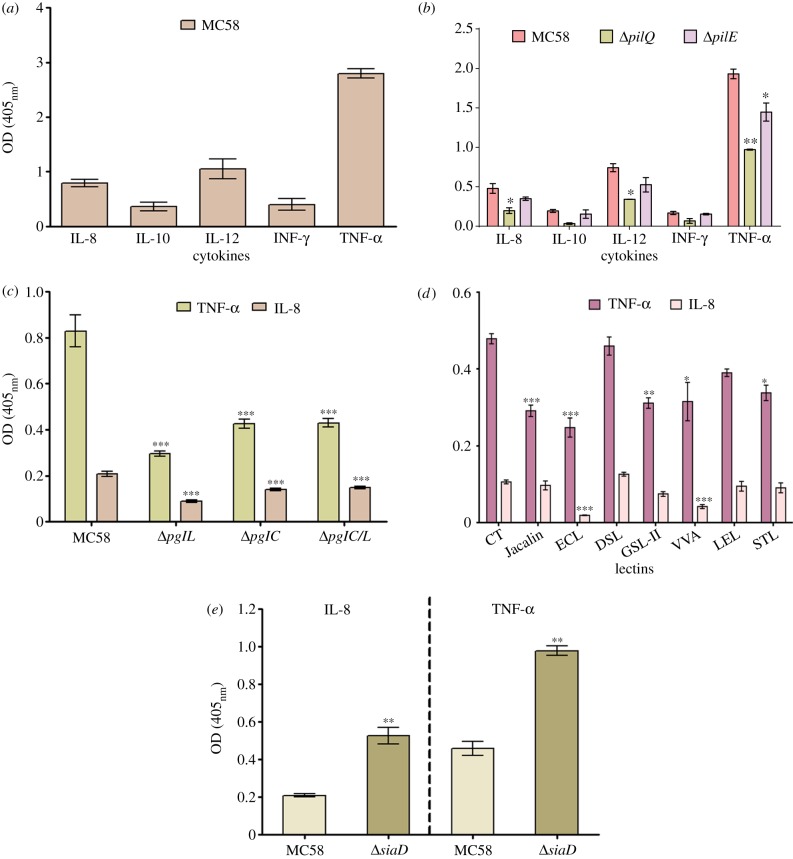
Binding of *Nm* to human cytokines. To determine the range and specificity of human cytokines that bind *Nm* strain MC58*,* IL-8, IL-10, IL-12, INF-γ and TNF-α were immobilized in amino-linked ELISA plates (bovine serum albumin was used as a negative control) and incubated with log-phase digoxigenin-labelled bacteria. (*a*) The wild-type strain MC58 bound to all the cytokines; the degree of binding varied marginally between the cytokines, except for TNF-α which was substantially higher. Data shown represent the results of four repeated ELISA tests. Error bars represent the mean ± s.e.m. of the absorbance values. (*b*) The binding of wild-type and mutant strains (Δ*pilQ* and Δ*pilE*) was examined in the same manner. Binding of Δ*pilE* and Δ*pilQ* to all of the examined cytokines was significantly reduced compared with the wild-type strain (*p* < 0.05, *t*-test), with the exception of the binding of Δ*pilE*/IL-12 (*p* > 0.05, *t*-test). Data shown represent the results of four repeated ELISA tests. Error bars represent the mean ± s.e.m. of the absorbance values. Binding of *Nm* to human cytokines is glycan dependent. (*c*) Glycosyl-transferase-deficient strains (Δ*pglC*, Δ*pglL* and the Δ*pglL/C*) were examined to assess their binding to IL-8 and TNF-α. All mutants exhibited somewhat defective binding to TNF-α and IL-8. (*d*) Inhibition of *Nm* binding to human cytokines using specific lectins. The specificity of the interaction between cytokines (i.e. TNF-α and IL-8) and the surface structure of *Nm* was examined. To identify the role of glycosylated surface structures of *Nm*, the inhibition of IL-8- and TNF-binding was performed using a series of lectins with different specificities. ELISA results revealed a significant reduction in *Nm* binding to TNF-α compared with untreated *Nm* with all lectins examined except DSL and LEL. The results shown are expressed as means ± s.e.m. for three independent experiments performed in triplicate. The asterisks indicate *p-*values of <0.05, *t-*test. (*e*) The intensity of binding of *Nm* capsule-deficient Δ*siaD* mutant strain MC58 to human TNF-α and IL-8 cytokines was measured using ELISA. The level of binding of the non-capsulated mutant was significantly higher in comparison with the wild-type strain, with binding to bovine serum albumin used as a background value. This observation is consistent with a role of the capsule in masking outer-membrane structures, and suggests that the capsule and its polysaccharide structures are not involved in interactions with cytokines. The results shown are expressed as mean ± s.e.m. for three independent experiments performed in triplicate. Asterisks indicate *p-*values of <0.05, *t-*test.

To determine the range and specificity of cytokines that bind *Nm*, IL-8, IL-10, IL-12, IFN-γ and TNF-α were immobilized in amino-linked enzyme-linked immunosorbant assay (ELISA) plates and incubated with digoxigenin-labelled log-phase-grown *Nm* strain MC58. The bacterial cells bound to different cytokines with a somewhat variable degree of binding among cytokines (figure 1*a*). TNF-α exhibited the highest binding signal, with the rest of the cytokines exhibiting lower signals with marginal variations. We then examined the concentration-dependent effects of cytokines (using TNF-α and IL-8) on bacterial growth. Bacteria were incubated with TNF-α or IL-8, and the growth was measured for a 24 h period. The mean average results for non-induced and cytokine-induced bacteria, collated from three independent experiments, show that TNF-α or IL-8 had no effect on bacterial growth (see electronic supplementary material, §S1*a*).

Our findings reveal an interaction between surface structures of the capsulated *Nm* serogroup B MC58 with TNF-α and IL-8. To examine whether this interaction is confined to *Nm* serogroup B or extends to other serogroups (different types of capsule structure classify *Nm* into different serogroups), five clinical isolates of serogroups B and Y were selected [28], and binding to TNF-α and IL-8 was assessed by ELISA. In all isolates from the two serogroups, the level of binding to TNF-α or IL-8 was comparable to the MC58 strain (see electronic supplementary material, §S1*b*).

A retagging technique [29] was used for the identification and purification of *Nm* TNF-α-binding adhesin(s); two subunits of the meningococcal Tfp, PilE (accession: P05431; 18.1 kDa; score: 1185.07) and PilQ (accession: Q70M91; 82.4 kDa; score: 1915), were identified as putative human cytokine receptors. To evaluate the contribution of these adhesins to bacterial binding, Δ*pilQ* and Δ*pilE* MC58 mutants were generated. The Δ*pilQ* mutation resulted in a significant reduction in binding to all of the examined cytokines (figure 1*b*). However, the Δ*pilE* mutation did not result in any significant reduction to cytokine binding with the exception of TNF-α (figure 1*b*). These data suggest that PilQ is an important meningococcal receptor for certain cytokines. However, it was impossible to confirm the important role of PilQ in a complemented strain in Δ*pilQ* mutant background because of a well-established transformation defect of this mutant. Several attempts using different transformation approaches were unsuccessful in introducing the complementing plasmid within the Δ*pilQ* mutant strain (data not shown).

### 3.2. Glycosylation of pili is crucial for binding to human cytokines

Many cytokines possess lectin-like activity that may be essential for the expression of their full biological activities. For example, pili are post-translationally modified (see electronic supplementary material, S2) and the surface accessibility of phosphorylcholine on pili is affected by changes to the structure of the pilin-linked glycan [15]. Here, we focused on the relevance of the lectin-like activity of cytokines in mediating *Nm* binding, using TNF-α and IL-8 as examples.

Three oligosaccharyltransferase (*O*-OTase) mutants (Δ*pglL*, Δ*pglC* and a double mutant) of the MC58 strain were generated and examined for binding to IL-8 and TNF-α. All three mutants exhibited significant reductions in binding to both cytokines (figure 1*c*). By contrast, a Δ*lgtF* mutant of the MC58 strain (defective for the synthesis of the polysialic acid capsule or LOS) [30] did not exhibit defective bacterial binding to IL-8 (see electronic supplementary material, figure S3).

To investigate further the nature of the interaction between cytokines and the surface structure of *Nm*, and to identify the role of glycosylated surface structures of *Nm*, the inhibition of IL-8- and TNF-α-binding were examined using a series of lectins—*Griffonia* (*Bandeiraea*) *simplicifolia* lectin II (GSL-II); *Datura stramonium* lectin (DSL); *Erythrina cristagalli* lectin (ECL); *Lycopersicon esculentum* (tomato) lectin (LEL); *Solanum tuberosum* (potato) lectin (STL); *Vicia villosa* agglutinin (VVA); *Artocarpus integrifolia* (*Jacalin*)—with different specificities (see electronic supplementary material, material and methods). The ELISA results revealed a significant reduction in *Nm* binding to TNF-α compared with untreated *Nm*, with all lectins examined except DSL and LEL (figure 1*d*). In addition, the binding of IL-8 was significantly reduced with ECL and VVA, suggesting that the IL-8 and TNF-α interactions are mediated by different PilE protein glycan moieties (figure 1*d*).

To exclude the role of the capsule in binding, a Δ*siaD* mutant (deficient in capsule formation) was generated, and the adhesion to TNF-α and IL-8 was examined. Binding of both cytokines to MC58 was significantly lower than that to the Δ*siaD* uncapsulated isogenic strain (figure 1*e*).

Collectively, the results of the lectin inhibition assays and the increased level of binding to cytokines in the Δ*siaD* uncapsulated and *O*-OTase *Nm* mutants suggest that the glycosylated structure of Tfp (pilin) plays a critical role in this interaction.

In addition, purified recombinant PilQ (expressed in *E. coli*, which possesses different glycosylation machinery than *Nm*) binds to IL-8 and TNF-α, indicating that it is unlikely to be glycosylated in the same manner as endogenous meningococcal PilQ. These data were consistent with our LC-MS/MS analysis, reinforcing the notion that PilQ is not glycosylated and providing additional evidence for a protein–protein interaction (see electronic supplementary material, §§S4 and S5).

### 3.3. IL-8 and TNF-α induce changes in *Nm* gene expression

**Figure 2.**
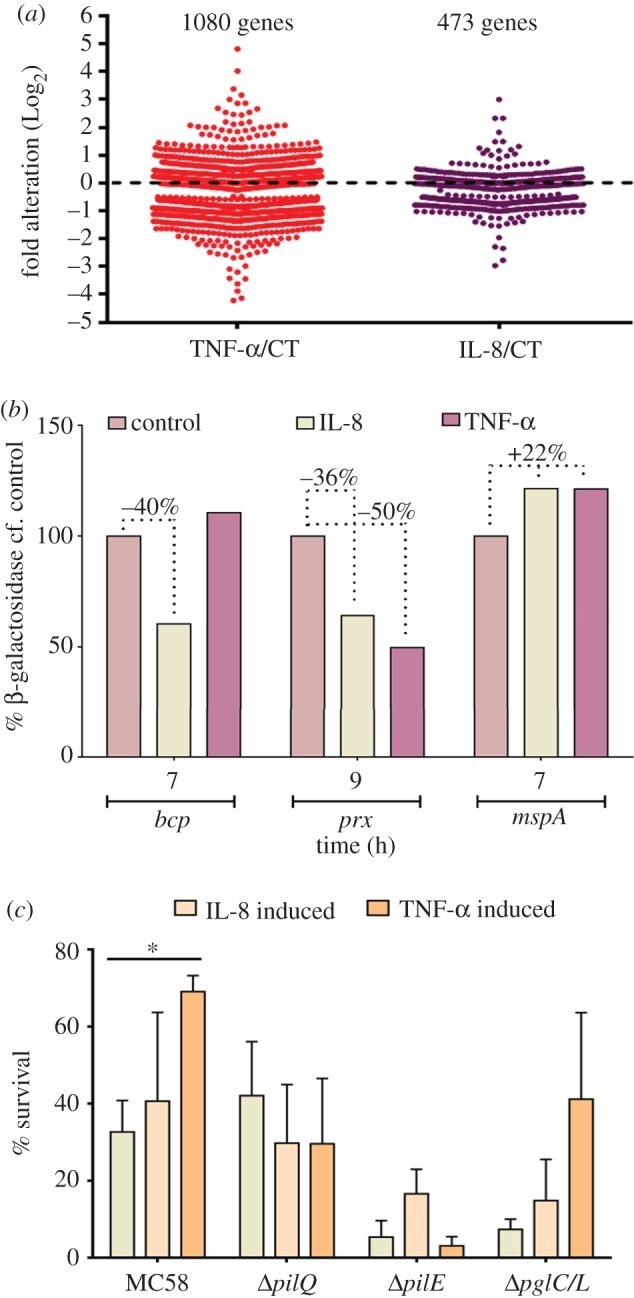
Effect of cytokines on *Nm* gene expression and resistance to complement-mediated lysis. (*a*) A general overview of transcriptome analysis from *Nm* induced at steady state with TNF-α or IL-8 and harvested at 9 h. (*b*) Reporter lacZ assays showing the expression of *bcp*, *prx* and *mspA* in cells induced with human cytokines at steady state and harvested at different time points (0, 4, 7, 9 and 12 h). The bar graphs show only the time points in which the corresponding gene expression was modulated at a maximum rate. Data were normalized against the untreated control (CT, control). A non-transformed wt MC58 strain was used as an additional negative control**.** (*c*) TNF-α- or IL-8-induced wt MC58 and the isogenic mutant strains were incubated with rabbit PilQ antibody followed by incubation with rabbit complement. TNF-α-induced wt MC58 bacteria survived significantly higher than mutant and non-induced bacteria. The results represent three independent experiments.

To investigate whether the bacterial interaction with human cytokines affects *Nm* gene expression, the transcriptional profiles were compared in non-induced and both IL-8 and TNF-α-induced cultures using deep-sequencing analysis. The RNA integrity of deep-sequencing samples was examined as explained in electronic supplementary material, §S6. In the IL-8 and TNF-α-induced cultures, the expression of 473 (approx. 20%) and 1080 (approx. 45%) *Nm* genes were altered, respectively (figure 2*a*; electronic supplementary material, §S7, separate Excel file (S_7_)). The genes with modified expression were categorized using the Genome Properties System (http://cmr.jcvi.org/tigr-scripts/CMR/CmrHomePage.cgi). A Log_2_ ratio of read counts higher than 0.5 was considered to indicate significant differences between induced and non-induced conditions.

Among the known genes, the expression of those coding for adhesion (**S_7_._1_**), energy metabolism (**S_7_._2_**), transport and binding proteins (**S_7_._3_**), cell envelope (**S_7_._4_**), bacterial survival (**S_7_._5_**), regulatory function (**S_7_._6_**) and amino acid synthesis (**S_7_._7_**) were altered. A significant portion of the regulated genes were of unknown function (i.e*.* hypothetical genes; electronic supplementary material, §S7, separate Excel file and Deep seq figures).

The comparison of the gene activation profiles in *Nm* led to the identification of genes that were down- or upregulated in cytokine-treated organisms and genes that were inversely regulated, such as those encoding PilE, cytochrome *c*, Iron ABC transporter, H.8 outer-membrane protein, MarR (AraC-family transcription regulator, *A*dhesion and *P*enetration *P*rotein (APP) [31] and *M*eningococcal *S*erine *P*rotease A (MSPA) [32] (electronic supplementary material, §S7, separate Excel file). Our study also showed that *SiaD* (NMB0067) and *Lip*A (NMB0082), which are involved in LPS production, are highly upregulated by TNF-α exposure.

All these genes probably play an important role in virulence and pathogenicity once bacteria are exposed to hyperinflammatory conditions. Furthermore, the prominence of regulated surface proteins and/or structures in the cytokine-treated bacteria indicates that host-secreted cytokines induce a profound remodelling of the bacterial cell membrane.

### 3.4. The effect of human cytokines on *Nm* gene expression using the reporter gene *lacZ*

In batch culture, it is unlikely that the level of expression at the beginning of growth is the same as the steady-state level [33], because the cultures are usually inoculated with cells from the stationary growth phase (‘overnights’) or with ‘uninduced’ cells. In the latter case, the β-galactosidase concentration in the inoculums was zero. Therefore, β-galactosidase activity was assayed using the method of Miller [34], after growth of *Nm* strains for up to 9 h (middle of log-phase) in DMEM in the presence or the absence of cytokines IL-8 or TNF-α (both 100 ng ml^−1^). The β-galactosidase activity measurements from strains of *Nm* bearing the promoter NMB0750 (bacterioferritin co-migratory protein, *bcp*), NMB0946 (peroxiredoxin, *prx*) and NMB1998 (*mspA*)-*lacZ* fusions confirmed that gene expression of *prx* and *bcp* is significantly regulated following the treatment with cytokines (figure 2*b*). The expression of *mspA*, however, was increased by 22% compared with uninduced bacteria.

### 3.5. Cytokines-induced *Nm* resistance to complement-mediated lysis

Resistance to complement-mediated lysis is a key virulence attribute of *Nm*. Meningococcus displays a vast repertoire of genes, often involved in virulence, whose expression is controlled by inflammatory cytokines mediated through reversible changes in the Tfp structure. The phenotypes observed here can therefore be due to variation in expression of genes involved in *Nm* surface structure. A functional study was carried out to validate the deep-sequencing data and differences in the transcriptional profiles observed to assess their role in *Nm* pathogenicity and/or virulence. To assess whether different cytokine treatments of wild-type (wt) MC58 and its isogenic mutants differ in sensitivity towards bactericidal activity, experiments were performed at the initial log-phase (9 h) of the *Nm* growth in the presence and the absence of cytokines (figure 2*c*). The rabbit antibody against recombinant PilQ in the presence of rabbit complement was capable of inducing 67% cell death in the untreated wt MC58 strain (i.e. 33% survived), whereas in the case of TNF-α-induced bacteria 31% cell death was induced (i.e. 69% survived). This is equivalent to a doubling of survival. By comparison, in the isogenic (Δ*pilQ*, Δ*pilE* and Δ*pglC/L*) mutants, there was no significant difference between cytokine-treated and non-treated bacteria. IL-8-treated bacteria did not exhibit any significant resistance to complement-mediated lysis (figure 2*c*). These data confirm the significance of modulation of surface structures (hence pathogenicity) and validate the results from deep sequencing.

### 3.6. *Nm* can ingest human cytokines, *in vivo*

**Figure 3.**
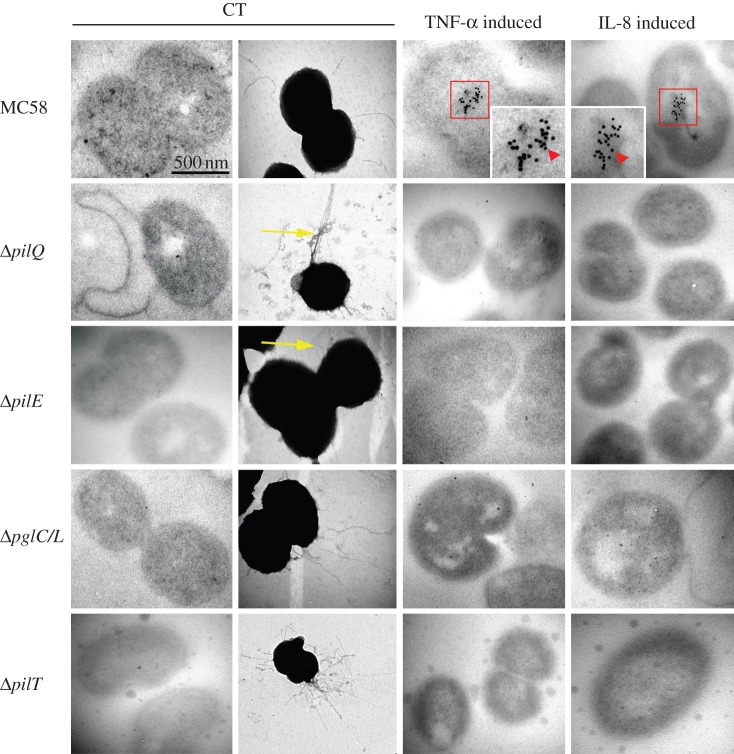
Transmission electron micrographs showing uptake of human cytokines by *Nm*. TEM analyses of TNF-α- or IL-8-induced *Nm* wild-type strain and related mutants cultured for 9 h in the presence or the absence of 40 ng ml^−1^ TNF-α or IL-8. Untreated bacterial strains used as a negative control (CT). TNF-α-induced wild-type shows clear accumulation of gold particles inside the bacteria. The cells shown in this image are representative of approximately 72% of the analysed bacterial population (see electronic supplementary material, material and methods). IL-8-induced bacteria exhibit clear accumulation of intracellular gold particles. No uptake was observed in images of TNF-α- or IL-8-induced Δ*pilE*, Δ*pilT* or Δ*pglC/L* mutants, indicating that uptake of cytokines specifically require the glycosylated form of PilE or retracting pili. A small percentage (less than 0.1% of studied population) of the PilQ-deficient bacterial population could ingest the TNF-α or IL-8 in periplasmic space. Negative staining of *ΔpglC/L* glycosyl-transferase confirmed that Tfp formation in the mutant strain was equivalent to the wild-type strain. The yellow arrow shows the formation of an unknown form of pili by *ΔpilE* and *ΔpilQ* mutants, which may be functional to some degree. To confirm the specificity of anti-IL-8 or anti-TNF-α, the TNF-α-induced bacterial cells were detected with anti-IL-8 and vice versa, and with secondary antibody. No gold particles were detected in all examined strains and this was considered as an additional definitive negative control. Three independent experiments carried out in the presence and the absence of human recombinant cytokines are shown. The scale bar represents 500 nm and the insets represent 2.5 times magnification.

To analyse the intracellular uptake of cytokines by *Nm*, wild-type bacteria and related mutants (Δ*pilQ*, Δ*pilE* and Δ*pglC/L*) were grown in DMEM and induced with either TNF-α or IL-8 (40 ng ml^−1^) for 9 h at 37°C. Cells were then extensively washed and fixed for transmission electron microscopy. The cytokines were detected with monoclonal antibodies, followed by secondary antibody conjugated with 10 nm colloidal gold particles. The majority of the TNF-α and IL-8-gold labelling was localized in the cytoplasm of wt *Nm* (figure 3, red arrowhead).

No TNF-α or IL-8 uptake was seen in the cytoplasm of the Δ*pilQ*, Δ*pilE*, Δ*pilT* or Δ*pglC/L* mutants. Mutations in the *pgl* or *pilT* (lacking pilus retraction ability) had no effect on normal piliation phenotype [35], also confirmed by this study (figure 3).

**Figure 4.**
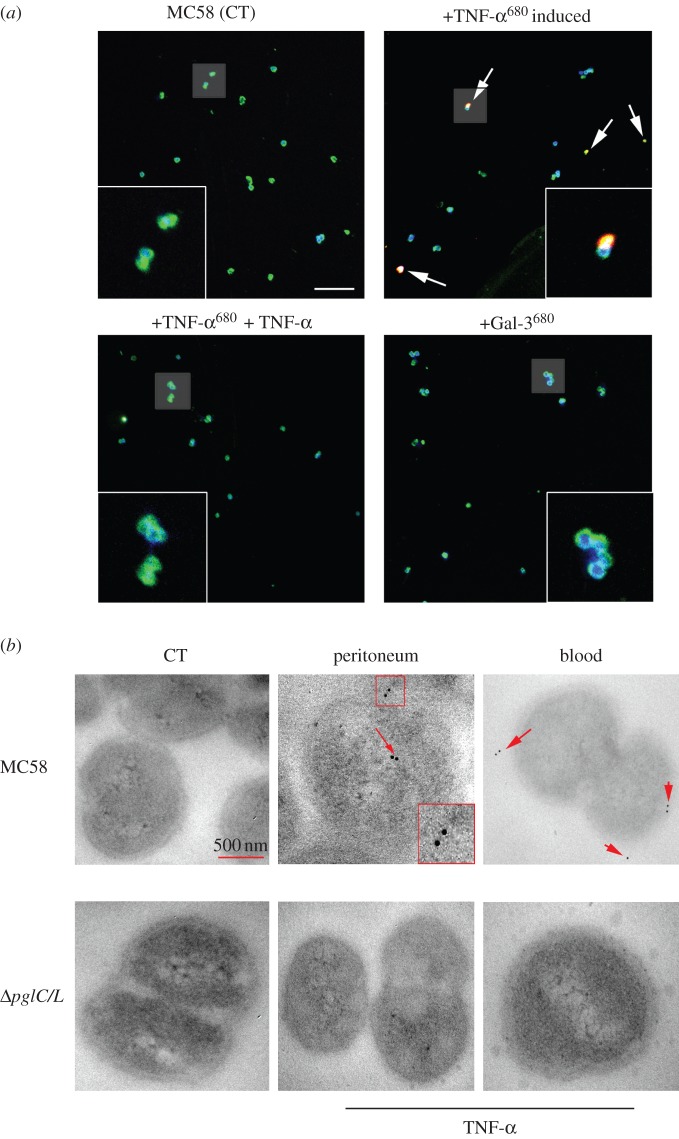
Confocal images of *Nm* uptake of human cytokines. (*a*) Detection of intracellular TNF-α into the meningococcal cytoplasm. *Nm* live cells were induced with Atto_680_-labelled recombinant proteins (TNF-α or Galactin-3). Cells were stained with anti-PorA monoclonal antibody and DPI. Merged images depict co-localization of Atto_680_ (red) and monoclonal anti-PorA antibody (green), DPI (blue) and +TNF-α^680^ induced (yellow). Non-induced *Nm* MC58 (CT) or Gal-3^680^ labelled was used as negative control. In addition, the uptake of labelled TNF-α^680^ was inhibited by using non-labelled TNF-α. Insets shown are at 2.5 times magnification. Images are single sections (300 nm) and data were collected from different fluorophores in separate channels. (*b*) Transmission electron micrograph of *Nm* TNF-α uptake in mice peritoneum and blood. TEM macrograph analysis of TNF-α uptake by *Nm* wild-type strain and Δ*pglC/L* mutant isolated from peritoneum and blood at 4 h post-infected mice. Treated samples with only secondary antibody were used as negative control (CT). Wild-type MC58 strain shows accumulation of gold particles inside or on the surface of the bacteria (red arrow). The cells shown in this image are representative of approximately 66% and 50% of the analysed bacterial population in peritoneum and blood, respectively. No uptake was observed in images of TNF-α Δ*pglC/L* mutants, indicating that uptake of cytokines requires the glycosylated form of PilE protein. The scale bar represents 500 nm and the insets represent two times magnification.

The intracellular uptake of labelled TNF-α by *Nm* wild-type MC58 bacteria was confirmed by confocal imaging, whereas in control experiments there was no uptake of labelled Gal-3 (galectin 3), confirming the specificity of the uptake (figure 4*a*). Furthermore, pre-treatment of the bacteria with non-labelled TNF-α inhibited the uptake of labelled TNF-α (figure 4*a*), further confirming uptake specificity.

In addition, MC58 *Nm* isolated directly from the peritoneum and blood of mice 4 h post-infection exhibited TNF-α uptake, whereas Δ*pglC/L* mutant *Nm* did not in EM experiments (figure 4*b*). Immunogold staining demonstrated 66% and 50% of the wt MC58 bacterial population in peritoneum and blood, respectively, showing uptake of TNF-α, but none of the examined samples from the Δ*pglC/L* mutant showed any signs of TNF-α binding and/or uptake. Collectively, our data confirmed the specific uptake of TNF-α *in vivo*.

### 3.7. Cytokines bind to the genomic DNA of *Nm in vivo*

We hypothesized that human cytokines may bind across the *Nm* genome, acting as transcription modulators for the expression of virulence genes such as autotransporter proteins MSPA and APP, in addition to the PptB transferase that adds phosphoglycerol onto Tfp [20]. Qualitative chromatin immunoprecipitation [36] was used to investigate the binding of cytokines to intergenic (promoter-containing) regions of these virulence genes.

**Figure 5.**
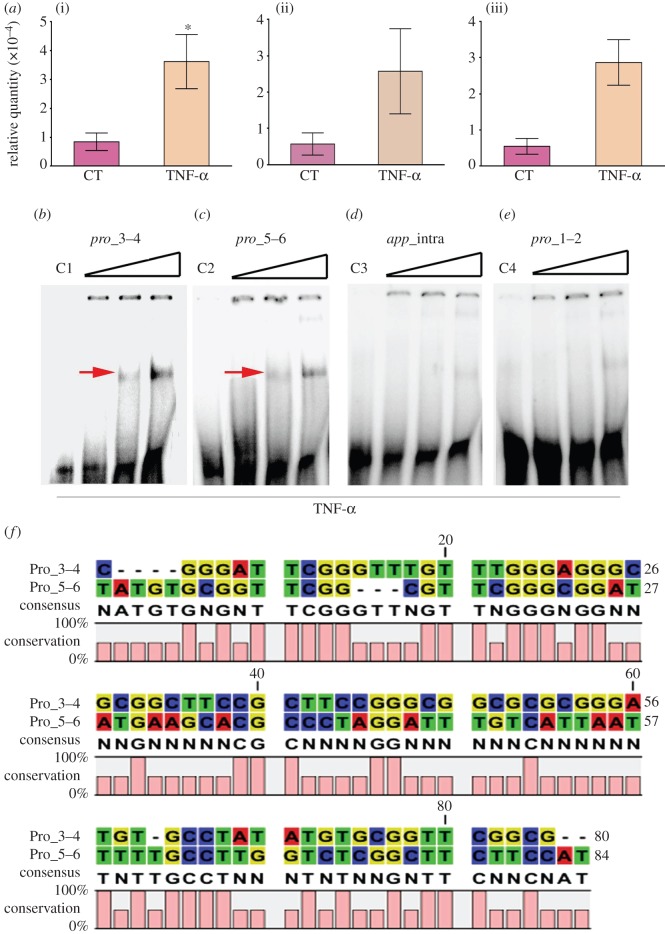
Chromatin immunoprecipitation. (*a*) An immunoprecipitation experiment to monitor the uptake and binding of TNF-α to the *Nm* genomic DNA. The wild-type strain MC58 was cultured and induced with TNF-α (100 µg ml^−1^) for 4 h. The panel depicts qPCR results, generated with primers designed to detect the intergenic regions of *app* or *mspA* and the open reading frame of *pptB* in each sample; (i) *app*, (ii) *mspA* and (iii) *pptB*. Five independent experiments are shown and non-induced strains were considered as negative controls. Binding of TNF-α to different *app* oligonucleotides of defined sequence was studied using a gel-shift assay. (*b*–*e*) Four DNA fragments (70–80 bp), three partially overlapping fragments from the intergenic region immediately upstream from the *app* gene (*b*,*c*,*e*) and one from the intragenic *app* gene region (*d*), were tested for TNF-α binding. Binding reactions were carried out at different concentrations of TNF-α (5, 10 and 20 μM). The control lane (DNA probe without protein present, 0 µM) contains a single band corresponding to the unbound DNA fragments (C1, C2, C3 and C4). Gel-shift complexes were resolved by electrophoresis and visualized by autoradiography. Red arrows indicate migration of the shifted TNF-α–DNA-binding complexes. (*f*) Alignment of two fragments from the intergenic region (*b* and *c*) suggesting potential DNA-sequences as TNF-α binding site(s).

The wt MC58 strain and the Δ*pilE*, Δ*pilQ* and Δ*pglC/L* mutants were grown in the presence or the absence of TNF-α, and antibodies directed against TNF-α were used to select protein cross-linked DNA fragments that were further purified using magnetic beads coated with a secondary antibody. Q-PCR was carried out using primers specific for the *app*, *mspA* and *pptB* promoter regions. The *app*, *pptB* and *mspA* immunoprecipitated DNA fragments from the TNF-α-induced wt strain were more than four- to fivefold enriched compared with the equivalent non-induced samples (figure 5*a*). These data confirm the uptake of TNF-α by wt *Nm* and suggest that TNF-α binds to genomic DNA sites within the *app*, *mspA* and *pptB* genes.

A gel-shift assay was used to study the direct binding of TNF-α to three partially overlapping fragments (70–84 bp) from the intergenic (promoter-containing) and one from the intragenic (non-promoter containing) regions of the *app* gene. Specific binding was detected with two fragments from the intergenic region immediately upstream from the *app* gene, but not with the other two fragments (figure 5*b–e*). The alignment of the two binding fragments suggests several conserved binding sites, which require further study to exquisitely define the precise sequence of the DNA-binding site (figure 5*f*).

### 3.8. Direct comparison of the mortality rates of wild-type and isogenic mutant *Nm* strains in an animal model

The pili of *Nm* interact with CD46, a human cell–surface protein involved in the regulation of complement activation. CD46, with the cytoplasmic tail Cyt-2, regulates T-cell-mediated inflammatory responses [37], is preferentially expressed in brain tissue and is tyrosine-phosphorylated by *Neisseria* [38]. The interaction with human CD46 represents a critical step for the onset of bacterial meningitis [39]. Transgenic mice expressing human CD46 are susceptible to meningococcal disease because bacteria can cross the blood–brain barrier in these mice [39].

**Figure 6.**
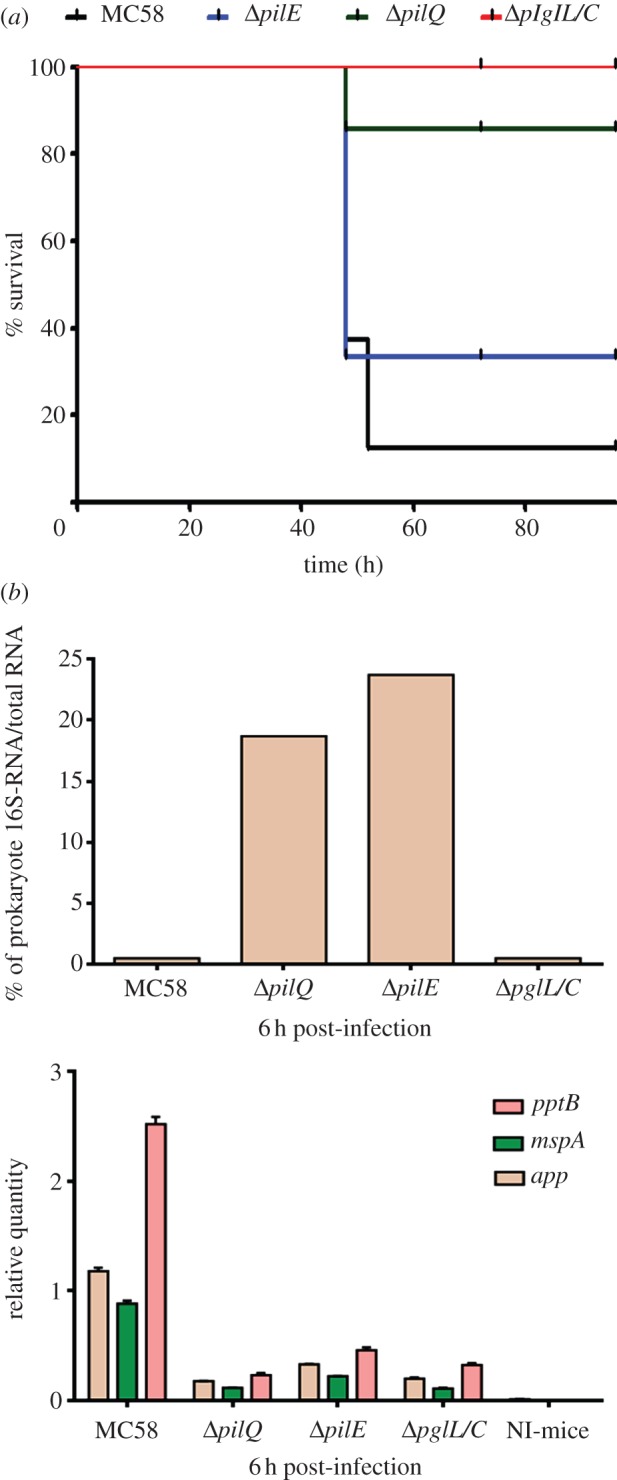
CD46 transgenic mouse model challenged with MC58, Δ*pilQ*, Δ*pilE* and Δ*pglC/L* mutants. (*a*) CD46 transgenic mice were intraperitoneally challenged (eight mice for each bacterial strain) with piliated *Nm* strain MC58 and isogenic mutant strains, and survival rates were assessed. The mortality rates were strain dependent. More apparent lethal disease occurred in mice that were challenged with MC58 and Δ*pilE*, whereas transgenic mice challenged with isogenic mutants (i.e. Δ*pglC/L* or Δ*pilQ*) survived significantly longer upon bacterial injection. (*b*) Relative quantification of *app*, *mspA* and *pptB* virulence genes. Blood samples were collected from mice infected with wt MC58 or the isogenic mutants at 6 h. The expression of examined genes in these samples was significantly higher in mice infected with the *Nm* MC58 strain than in mice infected with the isogenic mutant strains (lower panel). Non-infected (NI) mice were considered as negative control.

The impact of the *Nm* surface structures required for binding to human cytokines on bacteraemia and meningitis progression was investigated in CD46 transgenic mice. Eight mice in each group were intraperitoneally infected with wt or one of the isogenic mutants (Δ*pilQ*, Δ*pilE* and Δ*pglC/L*) and examined for 72 h. All of the mice developed bacteraemia (see electronic supplementary material, figure S9 and table S9), but lethal instances of disease occurred only in mice that were infected with MC58 or the Δ*pilE* mutant. Mice that were infected with Δ*pilQ* and Δ*pglC/L* mutants exhibited significantly less mortality and enhanced survival rates (figure 6*a*). These results further confirmed the importance of PilQ and surface glycans for *Nm* virulence.

In addition, blood samples were collected at 2, 6, 24 and 48 h post-infection to analyse the production of inflammatory cytokines such as TNF-α, IFN-γ, IL-6, keratinocyte chemoattractant (KC or CXCL-1, equivalent to human IL-8) and anti-inflammatory cytokine IL-10 (see electronic supplementary material, figure S9). The results showed that the Δ*pilQ* and Δ*pglC/L Nm* mutants can still activate immune cells to secrete inflammatory cytokines just like the wt strain. Collectively, our data suggest that although the *Nm* mutants can still activate cytokine secretion they are at the same time less responsive to cytokines (because they are lacking the specific adhesins); therefore, animal survival is enhanced.

Furthermore, the total RNA was purified from all collected blood (6 h post-infection) from each group of mice and pooled in one sample, in order to examine the expression of virulence genes such as *app*, *mspA* and *pptB* using Q-PCR. The results clearly showed that mice infected with wt MC58 were expressing these genes significantly higher than the isogenic mutants (figure 6*b*).

## 4. Discussion

*Nm* is usually a commensal bacterium of the nasopharynx. During the commensal state, most of the dividing bacteria belong to the same antigenic type and express low levels of virulence genes. Most probably, a peak of pathogenic status (bacteraemia followed by meningitis) is reached when the bacteria sense danger caused by a hyperinflamed environment. Here, we propose that host elicitors in response to microbes are engaged by commensal *Nm* and control the patterns of pathogenesis. Progression towards pathogenic states requires monitoring of long-term changes of bacterial behaviour to understand how this cycle varies across a broad inflammatory status. Our data provide novel insights into the changes taking place as inflammation continues to rise, and the extent and duration of inflammatory markers change.

TNF-α and IL-8 are pro-inflammatory cytokines that have numerous biological activities [40] and play important roles not only in host defence [40,41] but also in some of the pathological sequelae associated with various bacterial infections [42,43]. Here, we show that bacteria use human cytokines to sense the host environment.

*Nm* probably employs two mechanisms for binding to IL-8 and TNF-α. Cytokines contain lectin-like carbohydrate domains which are spatially distinct from cytokine-receptor binding sites [44]. The lectin-like domains of cytokines represent pathogen-specific recognition sites that can contribute to the elimination of pathogens via opsonization and/or leucocyte activation [44–46]. *Nm* might use this strategy to facilitate the neutralization and/or inactivation of human cytokines and the modulation of its own gene expression in response to environmental alterations.

Our data, with a series of glycosyl-transferase-deficient mutants (Δ*pglL*, Δ*pglC* and a Δ*pglC/L* double mutant), indeed suggest that *Nm*-cytokine binding is mediated partially by glycan moieties and by protein–protein interactions. This observation is consistent with the findings of Estabrook *et al*., who showed that mannose-binding lectin binds to the non-glycosylated *Nm* outer-membrane proteins Opa and PorB in a carbohydrate-independent manner [47]. Here, we show that binding of TNF-α or IL-8 to *Nm* is mediated by pilus assembly proteins (i.e. PilQ and PilE proteins) and that the virulence properties of *Nm* are enhanced as a consequence of TNF-α or IL-8 binding and uptake. The ingested cytokines directly bind to genomic DNA, and consequently regulate the expression of several genes.

TNF-α was shown to exhibit DNA-binding activity (figure 5*d,e*). This is consistent with a previous report showing that IL-8 binding to neutrophil receptors can be prevented by anionic polymers, such as DNA and actin [48]. The available crystal structures in the PDB (Protein Data Bank) show a single well-folded domain with mainly β sheets and no obvious DNA-binding motifs. It is not a requisite that all DNA-binding proteins possess standard recognized motifs, as several proteins can still bind DNA using other structural features.

Furthermore, we show that TNF-α is able to regulate bacterial gene expression. There is a variety of mechanisms that bacteria use to modulate gene expression in response to environmental stimuli. DNA binding by cytokines may change promoter architecture, which could then change the preference (negatively or positively) of RNA polymerase for certain promoters, physically prevent direct binding of RNA polymerase to promoters or prevent binding of transcription factors (gene enhancers or silencers). Indeed, many combinations of these mechanisms may work for different promoters of different virulent (and other) genes to affect global transcription. In addition, in bacteria there are several non-specific DNA-binding proteins, known as nucleoid-associated proteins (NAPs), that bind globally to the bacterial genome (nucleoid), and affect both its structure and its transcriptional activity at the global level (often by changing pervasive DNA superhelicity, which then affects gene expression, or bind directly at specific promoters working in concert with dedicated transcription factors to regulate gene expression in response to growth phase and environmental changes [49–51]. In one specific example from the literature, the off-to-on switching of cell-surface structures such as pyelonephritis-associated pili [52] in many enteric bacteria is environmentally regulated by a number of factors including the CpxAR two-component regulatory system, the Histone-like nucleoid structuring protein H-NS (a non-specific NAP) and cAMP-catabolite gene activator protein [53]. Hence, binding of cytokines to the bacterial genome could potentially elicit a variety of different structural/transcriptional global effects via any combination of the above mechanisms.

The fact that mice infected with the Δ*pglC/L* double mutant, expressing both PilQ and PilE proteins but lacking glycan moieties, developed significantly less disease, revealed its reduced virulence *in vivo*. The expression of virulence genes was still significantly higher in wt MC58-infected mice, showing that strains lacking pili appendages or glycans are less prone to adapt to the inflamed environment (i.e. less virulent). This was consistent with EM analysis of isolated bacteria from the peritoneum and blood samples of post-infected mice. Four hours post-infection inflammation is increased in the peritoneum and 66% of wt MC58 show TNF-α uptake compared with no uptake in the Δ*pglC/L* mutant (figure 4*b*). This is significant considering that *in vivo* many different cytokines could potentially interact with *Nm* in a complex manner, causing a wide range of changes in bacterial behaviour. Despite this complexity, we still see a considerable number of bacteria specifically interacting with TNF-α in the wt MC58 but not in the Δ*pglC/L* mutant isolated from infected animals.

However, no significant difference in bacterial levels and cytokine levels in the blood from infected mice were observed between the examined strains (see electronic supplementary material, §S9), showing that survival of the animals is not related to bacteraemia.

**Figure 7.**
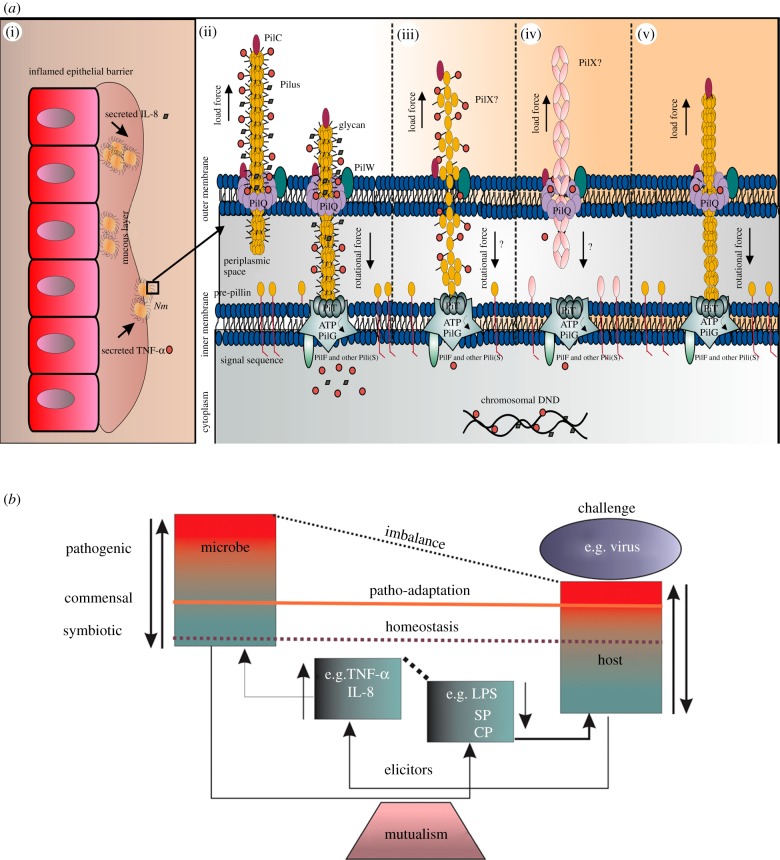
Illustration of phenotypic Tfp alterations in *Nm* mutants and their impact on the uptake of human cytokines. (*a*) Tfp mediates a vast array of functions, including adhesion, motility, microcolony formation, and secretion of proteases and colonization factors [54]. This functional versatility is probably a consequence of the capacity of Tfp to be retracted [55]. The rapid assembly/disassembly of Pili is mediated by PilT, a powerful molecular motor that hydrolyses ATP and plays an essential role in natural competence contributing to virulence by promoting an exquisite genetic adaptability. Another Tfp subunit PilW is an outer-membrane protein necessary for the stabilization and functionality of the fibres [56]. Our knowledge of Tfp function reveals a number of paradoxes. How does Tfp monitor the environment? How does a single filament explain its functional diversity? The schematic diagram explains the proposed mechanism of cytokine uptake using Tfp structures. Like many other bacteria, pathogenic *Neisseria* are detected by mucosal epithelial cells and sentinel immune cells in the epithelium. (i) Illustration of an epithelial barrier from the nasopharyngeal region colonized by *Nm*. The pathogenic *Neisseria* express a set of common virulence determinants that enable efficient colonization, immune evasion and transmission. Such functions reflect their high degree of adaptation to humans; rather than producing cytotoxins, these bacteria have evolved specialized mechanisms to promote growth and persistence in the host (reviewed in [57]). Furthermore, peptidoglycan fragments in outer-membrane vesicles are recognized by NOD-like (Nucleotide Oligomerization Domain) receptors in the cytoplasm of epithelial cells [58] and promote the local release of IL-8, IL-6, TNF-α, IL-1β and other cytokines [59,60]. (ii) Tfp from a wild-type strain exhibits a natural occurrence of pili formation and retraction machinery. (iii) Formation of alternative pili in a ΔpilQ mutant. A study by Wolfgang *et al*. [61] using TEM showed that the membranous protrusions in PilQ/T mutants in *N. gonorrhoea* contained pilus fibres. The pilus fibres were indistinguishable in diameter and morphology from those observed on the surface of wild-type cells, leading to the conclusion that PilQ is non-essential for pilus fibre formation and functions specifically in Tfp biogenesis by facilitating the translocation of the fibre to the cell surface [61]. (iv) Δ*pilE* mutant lacking Tfp pili. However, in absence of PilE, projection of PilX (unknown pili) results in unusual pili, which may not be fully functional as Tfp, but may still be somewhat responsive to environmental alterations through the PilQ protein. (v) Non-glycosylated Tfp on a Δ*pglL* mutant. This strain produces non-glycosylated pilin variants. (*b*) Tipping the balance towards inflammatory disease. A proposed homoeostatic mechanism showing the delicate balance between host and microbe that determines commensalism or virulence in response to the environment. The immune system is conventionally viewed as a means of fighting infection. It has become clear, however, that what is considered as the ‘immune’ system has also evolved to maintain homoeostasis and regulate commensal microbes that normally inhabit the body [62]. The shared evolutionary fate of humans and their symbiotic bacteria resulted in mutualistic interactions that are essential for human health [63]. Therefore, the recognition of the immune elicitors by bacterial cells provides a key to understanding disease processes. We propose that host responses to microbes are engaged by commensal *Nm* and control the patterns of pathogenesis. This hypothesis broadly serves as the conceptual foundation for understanding the interrelationships between microbes and humans. Symbiotic bacteria typically do not cause serious health problems to the host [64] and live in an equilibrated environment. This asymptomatic presence can even be beneficial to the human host because it allows antibodies to develop which can be useful in fighting a more serious subsequent infection [64]. However, *Nm* can sense (and respond to) the threat caused by an overreacted immune response to other pathogens, such as virus infections that can destabilize the balanced interrelationship between bacteria and host. Symbiotic bacteria either have degenerate genomes that retain only the most essential functions [65] and lack genes required for the ingestion of elicitors or eliminate the genes that proceed in response to host elicitors, thus maintaining the symbiotic interaction within their host. Our discovery paves the way to investigate novel ecological and evolutionary principles that may provide new strategies for restoring and maintaining human health. LPS, lipopolysaccharide; SP, secreted bacterial proteins; CP, cell envelope proteins.

In the case of the Δ*pilE* mutant, no gold particle uptake was observed (i.e. no cytokine uptake; figure 3), but the survival of infected mice was only 32% (figure 6*a*). However, the negative staining indicated that there was another form of pili-like surface appendages in the Δ*pilE* mutant, which could potentially be an alternative entry point for cytokines *in vivo*, which could affect survival (illustrated in figure 7*a*).

The Tfp system is widely distributed across many different bacteria species (more than 150 species), which suggests that cytokine entry into bacteria may be of wide applicability across many species. The binding of *Pseudomonas aeruginosa*, *Helicobacter pylori* and entero-pathogenic *E. coli* to a broad range of human cytokines was studied in parallel and the corresponding adhesins were identified (RM Delahay, H Wilkerson, P-J Royer, T Self, J Stoof, K Kong, I Notingher, P Soultanas, DAA Ala'Aldeen, J Mahdavi 2013, unpublished data). However, the detailed mechanisms of how these opportunistic pathogens actively sense alterations in the host and respond by enhancing their virulence phenotype remain to be studied.

Taken together, the proposed mechanism potentially has general applicability and the fundamental knowledge gained from investigations of the uptake system of this bacterium with biochemical analyses should yield substantial insights that will help to unlock more of the secrets of the lifestyle and pathogenic potential of this organism and, potentially, other pathogenic bacteria (illustrated in figure 7*b*). Our findings also provide a mechanism to explain the frequent development of meningitis in patients with an intense and protracted inflammatory response. Further research comparing the nature of hypervirulent lineages may elucidate the extent to which this feature contributes to the epidemiological distinctiveness of meningococcal infections.

In the future, our approach will focus on the identification of compounds structurally similar to cytokines that modulate the expression of bacterial virulence genes and reprogramme the bacterial behaviour towards less pathogenic and more commensal states. This knowledge could be directed towards the study of drugs and existing inhibitors, such as lectins, to assess their suitability as novel therapeutic agents. By exploiting knowledge of their modes and sites of action to combat bacterial infections, we could develop powerful novel arsenals against the serious problem of antibiotic resistance.

## 5. Material and methods

### 5.1. Enzyme-linked immunosorbant assay

Washes were performed at room temperature in PBS/T (0.1%) (phosphate-buffered saline/tween).

Purified, human recombinant IL-10, IL-12, IL-8 (aa 1–77), TNF-α (aa 77–233), (Super family, Member 2) and INF-γ (3 µg ml^−1^) (Sino Biological Inc, Beijing, China) were coupled to 96-well plates by adding 100 µl of cytokine solution (3 µg ml^−1^) diluted in sodium carbonate buffer (pH = 9) to each well. The bacterial strains MC58, Δ*pilQ*, and Δ*pilE*, Δ*pglC/L* were labelled with 10 µg ml^−1^ digoxigenin in PBS for 2 h at room temperature. A total of 100 µl of labelled bacteria (OD_600_ 0.02) were added to each well, and the plates were incubated overnight at 4°C.

The plate was incubated with a polyclonal anti-digoxigenin (alkaline phosphatase, 1 : 5000) in 1% BSA/PBS. Then, 100 µl of ABTS solution (5 mg ml^−1^; Roche) was added to each well. The absorbance at 405_nm_ was measured at different time points. For inhibition assay, the bacterial cells was pre-incubated with a series of lectins (see the electronic supplementary material, material and methods) for 2 h followed by several washes, then the bacterial cells were added to coated plates with various cytokines. The following steps were similar to ELISA.

### 5.2. Immunogold labelling and electron microscopy

Wild-type and mutants (Δ*pilQ*, Δ*pilE* and Δ*pg1L/C*) *Nm* were treated with either TNF-α or IL-8 for 4–9 h, with untreated replicates included for each strain/treatment combination. Bacteria were then fixed in 3% paraformaldehyde and 0.1% glutaraldehyde in phosphate buffer for 2 h at room temperature and processed for transmission electron microscopy. The samples were subsequently washed, dehydrated and processed into Araldite resin blocks (TAAB Laboratories), before being sectioned and mounted onto nickel grids. For the immunogold labelling, samples were washed (in a 1% BSA and 5% goat serum solution) and incubated overnight at 4°C with either anti-TNF-α or anti-IL-8 monoclonal antibodies at 2 µg ml^−1^ (Thermo Scientific), followed by labelling with goat anti-mouse IgG : 10 nm gold (BB International) at 0.2 µg ml^−1^ for 4 h at 4°C. To prevent non-specific binding with both primary and secondary antibodies, BSA and goat serum were used at appropriate stages in the procedure. Imaging was performed on an FEI Technai 12 Biotwin transmission electron microscope at 100 kV.

### 5.3. Chromatin IP

The IP protocol used in this study was the same as described in Grainger *et al*. with slight modifications [66,67].

### 5.4. DNA-binding studies

Electrophoretic mobility shift assays (EMSAs) were performed essentially as described [68]. Briefly, synthetic oligonucleotides from the *app* gene (NMB1985 APP) and its promoter region were isolated by PCR. Overlapping fragments used here are: Pro.1–2: TTT CGG TTG TCC GTT TGT CGG TTG TTT TCA TTA TTT TTC CTT ATC TGA CGG GAT TCG GGT TTG TTT GGG AT, Pro. 3–4: CGG GAT TCG GGT TTG TTT GGG AGG GCG CGG CTT CCG CTT CCG GGC GGC GCG CGG GAT GTG CCT ATA TGT GCG GTT CGG CG, Pro. 5–6: TAT GTG CGG TTC GGC GTT CGG GCG GAT ATG AAG CAC GCC CTA GGA TTT GTC ATT AAT TTT TGC CTT GGT CTC GGC TTC TTC CA, Internal region of *app* gene itself: GTT TTT TAC CAA CAG GAG GCT CAT TTG GCG ACA GTG GCT CAC CAA TGT TTA TCT ATG ATG CCC AAA AGC AAA AGT G. These were amplified and radioactively labelled using ^32^P-γATP and T_4_ polynucleotide kinase (New England Biolabs). Binding reactions were carried out with 50 000 cpm (approx. 0.1 ng) of ^32^P-end-labelled oligonucleotide for 20–30 min at room temperature in 10 or 20 μl reaction volumes containing 12% glycerol, 12 mM HEPES-NaOH (pH 7.9), 60 mM KCl, 5 mM MgCl_2,_ 4 mM Tris–Cl (pH 7.9), 0.6 mM EDTA (pH 7.9) and 0.6 mM dithiothreitol.

Protein–DNA complexes were resolved in 6% polyacrylamide gels pre-electrophoresed for 30 min at room temperature in 0.25 × TBE buffer (22.5 mM Tris borate and 0.5 mM EDTA, pH 8.3). Gels were dried and visualized using a phosphorimager.

### 5.5. Deep sequencing

Total RNA was enriched via two rounds of ribodepletion using a MICROBExpress kit (Ambion). Barcoded RNA-seq libraries were then constructed from each enriched RNA sample using a Total RNA-seq kit (Ambion) and sequenced using a SOLiD 4 genome analyser (Applied Biosystems).

The SOLiD reads were mapped to the reference genome (*Nm* MC58) using BioScope v. 1.3.1 software. The htseq-count script from the HTSeq Python package (http://www-huber.embl.de/users/anders/HTSeq/doc/count.html) was used to count the number of reads that were mapped to each gene. The total number of reads per kilobase per million mapped reads were also calculated [69]. Differential expression analysis between the samples was performed using the R package DEGseq [70].

### 5.6. Reporter gene *lacZ* assay

Promoter–*lacZ* fusions were constructed by inserting *Nm* promoter regions upstream of *lacZ*, creating promoter–lacZ translational fusions, using the *Bam*HI site upstream of *lacZ* in pLAS94 [71]. For gene NMB0946 (preroxiredoxin, *prx*), the promoter was amplified with primers 5′-AAAAGGATCCAGCACCCAAATCCACA-3′ and 5′ AAAAGGATCCCCGGTACGATCTTGCAAA-3′. For gene NMB0750 (bacterioferritin co-migratory protein, *bcp*), the promoter was amplified with primers 5′ AAAAGGATCCACATCCATAGTCCTAC-3’ and 5’ AAAAGGATCCTGTGAGGAGAGAATC-3′.

For gene NMB1998 (*mspA*), the promoter was amplified with 5′-CGCGGATCCCGCATGATGATTATCCGTGTA-3′ and 5’-CGCGGATCCAAC AACCGGAAAACGCAG-3′ by these sets of primers. Derivatives of pLES94 containing the promoter region were checked by sequencing and transformed into *Nm* MC58, and verified by PCR. β-galactosidase activity was assayed by the method of Miller [34], after growth of *Nm* strains for 0–12 h in DMEM medium in the presence or the absence of cytokines IL-8 or TNF-α, both at a concentration of 100 ng ml^−1^.

### 5.7. Mouse model of infection

Previous studies have shown that the hCD46Ge transgenic mouse line (CD46^+/+^) can develop meningococcal disease [39,72,73]. Serogroup B *Nm* MC58 and the mutant strains were suspended in GC liquid, and each mouse was challenged intraperitoneally (i.p.) with 1.2×10^9^ CFU in 100 μl of GC liquid medium. Experiments were performed with 6–8-week-old mice (*n* = 8 mice per group). In the control group, mice were challenged *i.p.* with 100 μl GC liquid. The health condition of all mice was closely monitored for 7 days. At the indicated time points, whole blood samples were collected from the tail vein for the measurement of cytokines, chemokines and bacteraemial levels.

Bacterial strains MC58 and Δ*pglC/L* mutant were isolated from blood of five *i.p.*-infected mice (1.2 × 10^9^ cfu mouse^−1^). Whole blood anticoagulated with heparan sulfate was collected by retro-orbital bleeding at 4 h post-infection. Blood cells were removed by gentle centrifugation; bacteria in plasma were washed once with PBS following fixation with 3% PFA, 0.1% glutaraldehyde in phosphate buffer.

## Supplementary Material

Pro-inflammatory cytokines can act as intracellular modulators of commensal bacterial virulence

## Supplementary Material

Deep sequencing raw data

## Supplementary Material

Deep Sequencing Figures
