# Supplementary material for: Pro-inflammatory cytokines can act as intracellular modulators of commensal bacterial virulence
Source: Open Biol. 2013 Oct;3(10):130048. doi: 10.1098/rsob.130048 (PMC3814720; doi:10.1098/rsob.130048)
Supplement: Pro-inflammatory cytokines can act as intracellular modulators of commensal bacterial virulence [file rsob130048supp1.docx]

**Supplementary online material**

**Pro-inflammatory cytokines can act as intracellular modulators of commensal bacterial virulence**

Jafar Mahdavi^1‡^, Pierre-Joseph Royer^1^, Hong S Sjölinder^7^, Sheyda Azimi^1^, Tim Self^2^, Jeroen Stoof^1^, Lee M. Wheldon^1^, Kristoffer Brännström^6^, Raymond Wilson^3^, Joanna Moreton^3^, James W. B. Moir^4^, Carina Sihlbom^5^, Thomas Borén^6^, Ann-Beth Jonsson^7^, Panos Soultanas^8‡^, **Dlawer A.A. Ala’Aldeen^1‡^**

##### **^1^**School of Molecular Medical Sciences, The University of Nottingham, Nottingham NG7 2RD, UK, **^2^**Institute of Cell Signaling, The University of Nottingham, Nottingham NG7 2UH, UK, **^3^**Deep Seq, Centre for Genetics and Genomics, The University of Nottingham, Queen's Medical Centre, Nottingham, NG7 2UH, UK, **^4^**Department of Biology, University of York, Heslington, York, YO10 5YW, UK. **^5^**Proteomics Core Facility, Sahlgrenska Academy, University of Gothenburg, Box 413, SE-405 30 Gothenburg, Sweden, **^6^**Department of Medical Biochemistry and Biophysics, Umeå University, SE-901 87 Umeå, Sweden. **^7^**Department of Genetic, Microbiology and Toxicology (GMT), Stockholm University, 109 61 Stockholm, Sweden, **^8^**School of Chemistry, The University of Nottingham, Nottingham NG7 2RD, UK.

^‡^ To whom correspondence should be addressed.

**Jafar Mahdavi**

Phone: +44-(0)115 8468925

Fax: +44-(0)115 823 0759

**E-mail:** [Jafar.Mahdavi@nottingham.ac.uk](mailto:Jafar.Mahdavi@nottingham.ac.uk)

**Panos Soultanas**

Phone: +44 (0)115 9513525

Fax: +44 (0)115 8468002

**E-mail:** [Panos.Soultanas@nottingham.ac.uk](mailto:Panos.Soultanas@nottingham.ac.uk)

**Dlawer Ala’Aldeen**

Phone: +44-(0)115 8230748

Fax: +44-(0)115 823 0759

**E-mail:** [daa@nottingham.ac.uk](mailto:daa@nottingham.ac.uk)

**Specific Discussion**

Over the course of infection by a pathogen, the immune system rapidly activates a number of defence mechanisms, characterized by the increased production of immune mediators (*i.e.,* cytokines). This reaction, known as the innate immune response, is mediated by pattern-recognition receptors that detect conserved structures found in a broad range of pathogens [[1](#_ENREF_1)]. *N. meningitidis* is usually a commensal bacterium of the nasopharynx. Factors that lead to the invasion of the bloodstream, often followed by the crossing of the blood-brain barrier and meningitis, are likely partly host- and partly bacterium-dependent. Hence, well-equilibrated gene regulation systems must exist, allowing the bacteria to monitor the environment and survive sufficiently long, without killing their host, to ensure an effective transmission of the species. During the commensal state, most of the dividing bacteria belong to the same antigenic type and express lower levels of virulence genes. Most likely, a peak of pathogenic status (*i.e.,* bacteraemia followed by meningitis) is reached when the bacteria sense danger caused by a hyper-inflamed environment (illustrated in **Fig. 7b,** main paper).

Tumor necrosis factor alpha (TNF-α) and IL-8 are pro-inflammatory cytokines that have numerous biological activities [[2](#_ENREF_2)] and are believed to play important roles not only in host defence [[2](#_ENREF_2),[3](#_ENREF_3)] but also in some of the pathological squeal associated with various bacterial infections [[4](#_ENREF_4),[5](#_ENREF_5)]. Consequently, bacteria developed sophisticated molecular machines for accurate sensing of the host environment and efficient uptake of host proteins which then modulate the expression of bacterial genes required for virulence and survival within the host.

Cytokines contain lectin-like carbohydrate domains which are spatially distinct from cytokine-receptor binding sites [[6](#_ENREF_6)]. Our investigation of the binding properties of a series of glycosyltransferase-deficient mutants (∆*pglC* and a ∆*pglC/L* double mutant) suggest that the *Nm*-cytokine binding is mediated partly by glycan moieties and by protein-protein interactions (**Fig. 2 main paper, S_3_-Fig.**). This observation is in agreement with the findings of Estabrook et al., who showed that mannose-binding lectin also binds to the non-glycosylated outer membrane proteins Opa and PorB of *Nm* in a carbohydrate-independent manner [[7](#_ENREF_7)]. The data presented here show that binding of TNF-α or IL-8 to *Nm* is mediated by pilus assembly (*i.e.,* PilQ and PilE proteins) and that the virulence properties of *Nm* are enhanced as a consequence of TNF-α or IL-8 binding and uptake (**Fig. 3** main paper). The ingested cytokines directly bind to genomic DNA (**Fig. 5** main paper) and consequently regulate the expression of several genes (**S_7_ and S_7_ Deep sequencing Figures**).

These findings have numerous implications in terms of our understanding of Tfp biogenesis/function and provide a useful groundwork for the precise functional characterization of the PilE, PilQ and other pili proteins.

*N. meningitidis* has evolved to become commensal within the nasopharyngeal epithelium. Once the bacterium comes into contact with the host epithelium, the program of gene modulation would remain more-or-less unaffected until substantial environmental variations occur. This implies a transient interaction with human epithelial cells and a tendency for the bacteria to reorganize their effectors and, consequently, transcription/translation profiles to rapidly adapt to new environmental conditions and, depending on the extent of environmental alterations, change towards a pathogenic status (**Illustrated in Fig. 7b main paper**).

In conclusion, our findings provide a mechanism to explain the frequent development of meningitis in patients with an intense and protracted inflammatory response. Further research comparing the nature of hypervirulent lineages may elucidate the extent to which this feature contributes to the epidemiological distinctiveness of meningococcal infections.

**Supplementary material 1a: The effect of human cytokines on *N. meningitidis* growth.**

Several studies have shown that pro-inflammatory cytokines enhance the growth of pathogenic bacteria such as *Pseudomonas aeruginosa*, *Staphylococcus aureus* and *Acinetobacter spp*. Meduri et al. provided additional evidence for a newly described pathogenic mechanism for bacterial proliferation in the presence of exaggerated and protracted inflammation [[8](#_ENREF_8)]. Here, we examined the effect of cytokines on the growth of *Nm* strain MC58. The results showed no changes in bacterial growth after incubation with either IL-8 or TNF-α (**S_1_**-Fig. a,b).

**b**

**a**

**S_1a_-Figures.** **Bacterial growth curves for *N. meningitidis.*** Bacterial growth in DMEM was determined by measuring the OD at 600 nm following incubation at 37^o^C, 5% CO_2_. Wild-type MC58 was either left untreated (CT) or treated with TNF-α (**a**) or IL-8 (**b**) at 20 or 40 ng/ml. All bacterial samples grew from an equal starting OD_600_ of 0.01. The time shown refers to the duration of the incubation and the marked 9 h time point (circle) refers to the harvesting time point for RNA purification.

**Supplementary material 1b: Binding of meningococcal clinical isolates to TNF-α and IL-8.**

To investigate the binding ability of other sero-groups of *N. meningitidis* strains (serogroups B and Y) to TNF-α and IL-8, 10 *N. meningitidis* clinical isolates were analysed in comparison with MC58 strain (serogroup B). The results clearly indicate that all meningococcal strains bind to both TNF-α and IL-8, albeit to variable degrees, irrespective of their invasiveness, phenotypic characteristics or geographical distribution (Fig. a, b).

**S1_b_-Figure: *N. meningitidis* clinical isolates bind to TNF-α and IL-8.** *N. meningitidis* strain MC58, and 5 clinical isolates from each serogroup (B and Y) were digoxigenin labeled and examined the interaction with TNF-α and IL-8 coated ELISA plate. All the strains were capable of binding to both TNF-α and IL-8, although at different degree. The data represent the mean (OD) at wavelength of 405 nm ± SEM (error bars) of a sample tested in triplicate. Experiments were repeated three times, with consistent results.

**Supplementary material 2. Glycosylation of PilE.** The *O*-linked glycans are associated covalently with the hydroxyl groups of serines or threonines. Type IV pilin (PilE) of pathogenic *Neisseria* was one of the first examples of an *O*-glycosylated glycoprotein [[9](#_ENREF_9)]. Two types of *O*-linked trisaccharide have been identified in *Nm* pili (the specific type expressed depending on the host strain): Gal-β1, 4-Gal-α1, 3-DATDH (DATDH represents 2, 4-diacetamido-2, 4, 6-trideoxyhexose), [[10](#_ENREF_10)] or Gal-β1, 4-Gal-α1, 3-GATDH (GATDH represents 2-glyceramido-4-acetamido-2, 4, 6-trideoxyhexose) [[11](#_ENREF_11)] (**S_2_**-Table ). An additional truncated *O*-linked disaccharide (Gal-α1, 3GlcNAc), is also present in *Nm* strain 8013 [[12](#_ENREF_12)].

| Protein target | | *O*-Tase transferase | | Amino acids modified | Glycan transferred | Ref. |
| --- | --- | --- | --- | --- | --- | --- |
| **Pilin (PilE)** | PglL | | Promiscuous; multiple *O*-linked repeats | Serine 63 |  | [[10](#_ENREF_10)] |
|  |  |  |  |  |  | [[11](#_ENREF_11)] |
|  |  |  |  |  |  | [[12](#_ENREF_12)] |

**S_2_**-**Table**: Structure of meningococcal *O*-linked glycosylated pilin. Adopted from [[13](#_ENREF_13)] *Key to symbols:

DATDH

G

ATDH

Gal

GlcNAc

A novel *O*-linked trisaccharide substituent, which has not previously been identified as a constituent of glycoproteins, is present within a peptide spanning amino acid residues 45 to 73 of the PilE molecule. This structure contains a terminal 1-4-linked digalactose moiety that is covalently linked to a 2,4-diacetamido-2,4,6-trideoxyhexose sugar, which is directly attached to pilin [[10](#_ENREF_10)].

Many cytokines possess lectin-like activity that may be essential for the expression of their full biological activities. Here, we focused on the relevance of the lectin-like activity of cytokines in mediating *Nm* binding, using TNF-α and IL-8 as illustrative examples.

It is well established that IL-8 and TNF-α interact with heparan sulfate proteoglycans [[14](#_ENREF_14)]. IL-8 has three binding domains: a high-affinity binding domain, a glycosaminoglycan-binding domain [[15](#_ENREF_15)], and another high-affinity binding domain located in the N-terminus of the cytokine [[16](#_ENREF_16),[17](#_ENREF_17)]. Previously, Fervert, et al., showed that the IL-8 glycosaminoglycan binding domain determines the location where IL-8 binds in lung tissue, a process mediated by heparan sulphate or chondroitin sulphate [[18](#_ENREF_18)].

**Supplementary material 3.** Three oligosaccharyltransferase (*O*-OTase) mutants (∆*pglL*, ∆*pglC* and a double mutant) of the MC58 strain were generated and examined for binding to IL-8 and TNF-α. All three mutants exhibited significant reductions in binding to both IL-8 and TNF-α (**S_3_-**Fig. and **Fig. 1c main paper)**. In contrast, a ∆*lgtF* mutant of the MC58 strain (defective for the synthesis of the polysialic acid capsule or the lipooligosaccharide; LOS), did not exhibit any defects in bacterial binding to IL-8 (**S_3_**-Fig.) [[19](#_ENREF_19)].

**S_3_-Figure. The binding of IL-8 to *N. meningitidis* is glycan mediated.**

Plates were coated with IL-8 and probed with DIG labeled bacteria. Strains that were deficient in *O*-glycotransferase (Δ*pglC*) exhibit less IL-8 binding. However, the binding of IL-8 to the Δ*lgtF* mutant was comparable to the WT strain. In addition, the Δ*pilQ* and Δ*pilE* mutants

*******

*****

**

were used for assessment. The values are representative of three independent experiments (*p*<0.0001, one-way ANOVA).

**Supplementary material 4. Recombinant PilQ binds to human cytokines.** To confirm PilQ specificity in binding to cytokines, the binding of purified recombinant PilQ (which is unlikely to be glycosylated in the same fashion as endogenous meningococcal PilQ), to cytokines was compared to purified recombinant PorA (an outer membrane protein). ELISA plates were coated with IL-8 and TNF-α and probed with either purified recombinant PorA or PilQ. The results revealed that recombinant PilQ binds significantly to IL-8 and TNF-α but only marginally to recombinant PorA (*p*< 0.0001, *t*-test).

**S_4_-Figure.** The purified recombinant PilQ was used for binding to cytokines in comparison to purified recombinant PorA. Only PilQ exhibited strong binding whilst PorA did not, suggesting a protein-protein interaction.

The results shown are expressed as means ± SEM for three independent experiments

carried out in triplicate. The asterisks indicate *P* values of <0.05, *t-*test.

**Supplementary material 5. Characterization of purified PilQ for possible glycosylation.** It has been previously shown that the PilQ subunit of Type IV pili plays an important role in the interaction between cytokines and bacteria. PilQ was purified from WT strain MC58 and analyzed by mass spectrometry (specifically, a nanoflow LC system coupled to a Orbitrap Velos). The sample was reduced and alkylated prior to digestion as previously described [[20](#_ENREF_20)]. After digestion, the samples were subjected to LC-MS/MS analysis using a hybrid linear ion trap-Orbitrap Velos mass spectrometer that was operated in data-dependent mode and automatically switched to MS/MS mode. MS-spectra were acquired in the Orbitrap, while MS/MS-spectra were acquired in the LTQ-trap. For each MS scan, the ten most intense, double, triple and quadruple-charged ions were sequentially fragmented in the linear trap by collision-induced dissociation. All tandem mass spectra were searched by Proteome Discoverer (Thermo Scientific, San Jose, CA, USA), incorporating MASCOT (Matrix Science, London, UK) against the SwissProt database (released 2011-06). The coverage of the analyzed peptides was 76% of the PilQ protein sequence, and none of the peptides indicated any post-translational modification by glycan insertion. All MS/MS spectra were also manually searched for diagnostic glycan ions without any match.

MNTKLTKIIS GLFVATAAFQ TASAGNITDI KVSSLPNKQK IVK**VSFDKEI VNPTGFVTSS PARIALDFEQ TGISMDQQVL EYADPLLSKI SAAQNSSR**AR

**LVLNLNKPGQ YNTEVR**GNK**V WIFINESDDT VSAPARPAVK** AAPAAPAK**QQ AAAPSTKSAV SVSEPFTPAK** **QQAAAPFTES VVSVSAPFSP AKQQAAASAK**

**QQAAAPAK**QQ AAAPAKQQAA APAK**QTNIDF R**KDGK**NAGII ELAALGFAGQ PDISQQHDHI IVTLKNHTLP TTLQRSLDVA DFK**TPVQKVT LK**RLNNDTQL**

**IITTAGNWEL VNKSAAPGYF TFQVLPKKQN LESGGVNNAP K**TFTGR**KISL DFQDVEIRTI LQILAKESGM NIVASDSVNG K**MTLSLK**DVP WDQALDLVMQ**

**AR**NLDMR**QQG NIVNIAPR**DE LLAKDKALLQ AEK**DIADLGA LYSQNFQLK**Y KNVEEFRSIL R**LDNADTTGN R**NTLISGR**GS VLIDPATNTL IVTDTR**SVIE

KFR**KLIDELD VPAQQVMIEA RIVEAADGFS R**DLGVKFGAT GKKK**LKNDTS AFGWGVNSGF GGDDKWGAET KINLPITAAA NSISLVRAIS SGALNLELSA**

**SESLSK**TKTL ANPRVLTQNR KEAK**IESGYE IPFTVTSIAN GGSSTNTELK KAVLGLTVTP NITPDGQIIM TVKINKDSPA QCASGNQTIL CISTKNLNTQ**

**AMVENGGTLI VGGIYEEDNG NTLTKVPLLG DIPVIGNLFK** TRGKKTDRR**E LLIFITPRIM GTAGNSLRY**.

**S_5_-Figure**. **Green**: high confidence; Yellow: moderate confidence; **Red**: low confidence.

**Supplementary material 6: RNA Integrity Assessment**

*Nm* strain MC58 was cultured in D-MEM medium at 40 ng/ml cytokines and samples were taken at different time points from 0 to 24 h of co-culture. Following putative induction of bacterial gene regulation with IL-8 or TNF-α, RNA samples were collected and purified from bacteria grown at different time points. The RNA Integrity Number (RIN) data (**S_6_-Fig. a**) indicated that


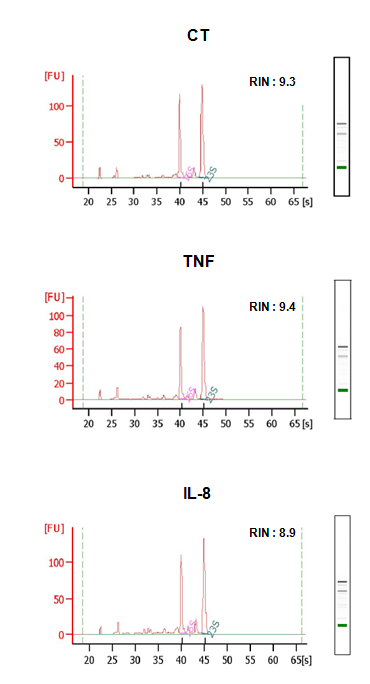


**b**

**a**

**S_6_-Figure. Determination of RNA integrity.** (**a**) Bacteria were cultured with or without TNF-α or IL-8 (40 *ng/ml*). RNA was then extracted at the indicated time points, and RNA integrity was measured using a Bio-Analyzer 2100 (Agilent). (**b**) Analysis of RNA integrity of deep sequencing samples. RNA integrity number (RIN) values are indicated on the graphs.

RNA quality decreased with prolonged incubation time and revealed that the optimal time point was 9 h (RIN˃9). Following incubation, bacterial RNA was isolated and purified. RNA purity and quality were measured using a Nanodrop 1000 Spectrophotometer and an Agilent 2100 Bioanalyser to obtain an *RNA Integrity Number* (RIN). The RIN data indicated that RNA quality decreased with prolonged bacterial culture times (**S_6_-Fig. b**). All samples (except those incubated for 24 h) presented RNA of a quality that was deemed acceptable (RIN ≥ 7).

**Supplementary material 7: Deep sequencing.**

Our results indicate that human cytokines (*i.e.* TNF-α and IL-8) may act either as specificity modulators of the RNA polymerase (RNAP) holoenzyme endowing an ability to recognize different promoters for differential gene expression [[21](#_ENREF_21)] or as *de novo* transcription factors. Transcrip­tion factors can either promote or repress gene expression, depending on the promoters with which they interact. *Nm* has a relatively small repertoire of transcription factors [[22](#_ENREF_22)] compared to *E. coli*, which encodes 314 transcription factors (of which 35% are activators, 43% are repressors and 22% are dual regulators) [[23](#_ENREF_23)]. However, *Nm* is equipped with a global regulator gene named NMB0573 (annotated AsnC), a member of the Lrp-AsnC family of regulators that are widely expressed in both bacteria and archaea. AsnC controls responses to nutrient availability through indicators of general amino acid abundance: leucine and methionine [[22](#_ENREF_22)]. Deep sequencing results revealed that this gene is up-regulated in TNF-α-induced *Nm* (**see separate supplementary Excel file**), indicating that TNF-α can modulate the expression of global regulatory genes and indirectly control genome-wide differential gene expression.

In addition to this fundamental regulatory machinery, vari­ous other proteins such as cytokines and regulatory elements can increase the complexity of the events leading from DNA to pro­tein: RNAP-associated proteins affect the processivity of RNAP [[24](#_ENREF_24)]; and internal promoters within operons [[25](#_ENREF_25)], small RNAs (sRNAs) [[26](#_ENREF_26)] and riboswitches (RNAs that regulate their own gene activity) [[27](#_ENREF_27)] affect transcription. The mechanism by which bacteria use cytokines to regulate transcription is likely to be complex and is still a mystery. The biological significance of other human cytokines on *Nm* remains to be thoroughly investigated.

Whole transcriptome analysis was also performed in both TNF-α and IL-8 treated cultures. In the IL-8 and TNF-α treated cultures, 473 and 1080 genes were identified as being differentially expressed in comparison to the control, respectively. Genes with a copy number lower than 50, for the three conditions used (CT, TNF-α, IL-8), were excluded from the analysis and only genes where the log_2_ ratio of the read counts was higher than 0.5 were discussed below.

A large proportion (**see separate supplementary Excel file**) of the regulated genes encoded proteins with unknown functions (*i.e*., hypothetical genes). Among the known genes, those coding for pili, capsule proteins and cell wall components, in addition to those involved in metabolism and the synthesis of proteins, nucleotides, LPS (lipopolysaccharide) and ATP, were modified in expression.

Collectively, these data suggest that TNF-α and IL-8 treatments induced a major signal for bacterial gene regulation.

**S_7-1_. *Adhesion***

In studying the biology of *Nm* invasion, a large number of studies have shown that after the first phase of localized adherence in which pili play an essential role, the genes of pili biosynthesis are regulated, following intimate attachment and diffuse adherence, or culturing *Nm* in human blood [[28](#_ENREF_28)].

Herein, our data show that a series of genes related to adhesion (including pili) and encoding proteins that mediate the interaction of Nm to eukaryotic cells were regulated to different extents after the induction of bacterial cells with IL-8 or TNF-α. In contrast, the regulation of these genes was independent of any cell association and/or attachment.

The pili genes involved in type IV pili biogenesis, for instance PilS (NMB0020), PilO (NMB1810), PilX (NMB0890), PilF (NMB0329), PilP (NMB1811), and PilE (NMB0018), were regulated in induced bacteria. Interestingly, IL-8 and TNF-α induction have opposite effects, indicating that different cytokines fill different functions from a bacterial viewpoint. For example, the expression of genes associated with type IV adhesion (*i.e.,* PilE [NMB0018] or PilQ [NMB1812]), whose products are involved in interactions with epithelial cells, were upregulated after induction with human TNF-α, but down-regulated in the case of IL-8 induction. We observed a similar pattern for *pilT* (NMB0052), which is responsible for pili retraction [[29-31](#_ENREF_29)], competence for DNA transformation [[30](#_ENREF_30)], and progression from the initial stage of adherence to diffuse adherence [[32](#_ENREF_32)].

In addition, intimate attachment requires the involvement of membrane-associated proteins that interact with specific cellular receptors. Several bacterial proteins have been proposed to fulfill this function, the best candidates being the Opc Class 5 outer membrane protein (NMB1053) and OpA (NMB1636) proteins, MafB (NMB2105) [[33](#_ENREF_33)], and adhesins (such as APP and MSPA NMB1985, MSPA; NMB1998), [[34](#_ENREF_34),[35](#_ENREF_35)]. Deep sequencing data showed that the *opc* and the adhesin genes were regulated during induction.

**S_7-2_. *Energy Metabolism***

It has been shown that exposure of *Nm* to various cytokines or immune cells has an impact on bacterial energy metabolism [[28](#_ENREF_28),[36](#_ENREF_36)]. This observation confirms the effect of environmental change on bacterial metabolism and survival. We noted a general downregulation in the expression of genes involved in energy metabolism following TNF-α and IL-8 treatment. However, the transcription of genes involved in energy metabolism, such as acetate kinase (NMB0435) and cytochrome C (NMB1887), are highly upregulated after TNF-α treatment, as reported after exposure to epithelial cells [[36](#_ENREF_36)] or human whole blood [[28](#_ENREF_28)].

**S_7-3_. *Transport and binding protein***

Bacterial secreted proteins play an important role in disease pathogenesis, as many of these proteins have toxin-like effects or act as adhesins that interact with host cells. In Gram negative bacteria, secretion proteins are more complicated than in gram positive ones because the proteins must cross the inner membrane, the peptidoglycan layer in the periplasmic space and the outer membrane.

Various secretion systems have been identified in gram negative bacteria, which are classified as Type I, II, III, IV, autotransporters or Two-Partner secretion systems (TPS), [[37](#_ENREF_37),[38](#_ENREF_38)].

Secretion through the inner and outer membranes may occur in two steps, in which a signal peptide targeting the Sec or Tat machinery enables the secreted protein to cross the inner membrane. Crossing of the outer membrane occurs through TPS, Type II secretion or autotransporter secretion pathways. Type I, III and IV secretion systems are dedicated protein complexes that secrete proteins across the two membranes in one step. *Nm* expressed both a Type I secretion system and a TPS [[38](#_ENREF_38)]. In this study, the treatment of *Nm* with TNF-α and IL-8 resulted in a massive downregulation of genes related to transport and binding proteins, such as ABC transporters (NMB0041, NMB0787, NMB1240, NMB1400, NMB1966, NMB 2026) and the amino acid ABC transporter. Similar effects have been observed after infection of epithelial cells by *Nm* [[36](#_ENREF_36)]. The other important group of transporter and binding proteins that are regulated are proteins that are involved in iron acquisition. It has already been shown that various iron sources have a major effect on meningococcal survival and regulate gene expression in *Nm* [[39](#_ENREF_39),[40](#_ENREF_40)]. Here, we found that treatment with TNF-α promoted an increase in expression of bacterioferritin B (NMB1206). A transcriptome study of *Nm* after 90 minutes of infection of whole human blood also revealed down-regulated expression of this gene [[28](#_ENREF_28),[41](#_ENREF_41)].

In addition, genes involved in sugar transport, such as the glucose/galactose transporter (NMB0535) and the outer membrane lipoprotein carrier protein (NMB0622), [[42](#_ENREF_42)], have been shown to be modulated in response to cytokine treatment.

**S_7-4_. *Cell envelope***

The modulation of cell-envelope gene expression was analyzed after the deep sequencing analysis. Treatment with TNF-α induced the modulation of 37 out of 48 genes. The modulation profile is balanced, with 18 genes upregulated and 19 genes downregulated. The impact of IL-8 treatment was less dramatic.

Remarkably, modulated genes included (as examples) polysialic acid capsule biosynthesis protein SiaD (NMB0067) ,which was upregulated after TNF-α treatment (log_2_ ratio + 2.19); lipid-A-disaccharide synthase (NMB0082), which is involved in LPS biosynthesis and was downregulated by both TNF-α and IL8 treatments; and penicillin binding protein (NMB0877) which was upregulated after TNF-α treatment.

**S_7-5_. *Bacterial survival***

Bacterial genes involved in capsule production are important for bacterial survival in various conditions during the infection and carrier stages [[43](#_ENREF_43)]. Genes such as *SiaD*, *SiaB* and *SiaC,* which are involved in sialic acid production, and sialyltransferase are important in terms of bacterial survival in blood. Our study showed that *SiaD* (NMB0067) and *Lip*A (NMB0082), which are involved in LPS production, are highly upregulated by TNF-α exposure.

Another important protein affected by cytokine induction is AniA (NMB 1623). This protein is involved in nitric oxidation and is conserved within various strains of Neisseria *ssp*. This protein is always expressed in various serogroups of *Nm*, apart from some carrier strains [[44-46](#_ENREF_44)]. Transcriptome analysis showed a decrease expression of AniA after IL-8 induction.

**S_7-6_: *Regulatory function***

Genes involved in expression of *fur* (ferric uptake regulation protein; NMB0205) or the GntR family transcriptional regulator (NMB1711) were dramatically repressed by TNF-α treatment.

In contrast, the MarR family transcriptional factor gene (NMB1585) and the mtrA AraC-family transcriptional regulator (NMB1591), a sequence specific DNA binding protein involved in regulation of antimicrobial efflux systems [[47](#_ENREF_47)], were upregulated after TNF-α treatment.

The protein hfq (Host Factor-I; NMB0748) is a RNA chaperone and key modulator of environmental stress responses [[28](#_ENREF_28),[48](#_ENREF_48)]. After IL-8 treatment, most of the genes were downregulated compared to control cultures, with the exception of *hfq*.

**S_7-7_. *Amino acid synthesis***

Sixteen genes with amino acid functions are presented here. The transcription of all 16 genes was decreased after IL-8 treatment. TNF-α treatment showed the highest effect on *hisB* imidazoleglycerol-phosphate dehydratase (NMB1583) and isopropylmalate isomerase- small subunit (NMB1034) by increasing the level of transcription. This study also showed that transcription genes such as cysteine synthase (NMB0763) and diaminopimelate epimerase (NMB0760), which is involved in lysine synthesis [[49](#_ENREF_49)] are downregulated in response to TNF-α treatment.

**Supplementary material 8: Chromatin immunoprecipitation (IP), *in vivo*.**

Three virulence factor genes were chosen for investigation: *app, mspA* and *pptB*. Qualitative IP was used to investigate the binding of cytokines to the promoter regions of the corresponding genes, *in vivo*. The ∆*pilQ*, ∆*pilE* and ∆*pglC/L* mutants was grown in the presence or absence of TNF-α, followed by formaldehyde crosslinking of nucleoprotein complexes, extraction of genomic DNA, and fragmentation via sonication to obtain approximately 500-bp DNA fragments. Antibodies directed against TNF-α were then used to select protein cross-linked DNA fragments that were further purified using magnetic beads coated with a secondary antibody. Subsequently, qPCR was performed using primers specific for *app*, *pptB* and *mspA*. The *app*, *pptB* and *mspA* immunoprecipitated DNA fragments from the TNF-α-induced ∆*pilQ* strain were more than 31-46-fold enriched, however not significant, compared to the equivalent samples from the non-induced ∆*pilQ* mutant. These data confirm the uptake of TNF-α by ∆*pilQ* mutant and again suggest that TNF-α binds to genomic DNA sites within the *app*, *mspA* and *pptB* genes (**S_8_-Fig.**).


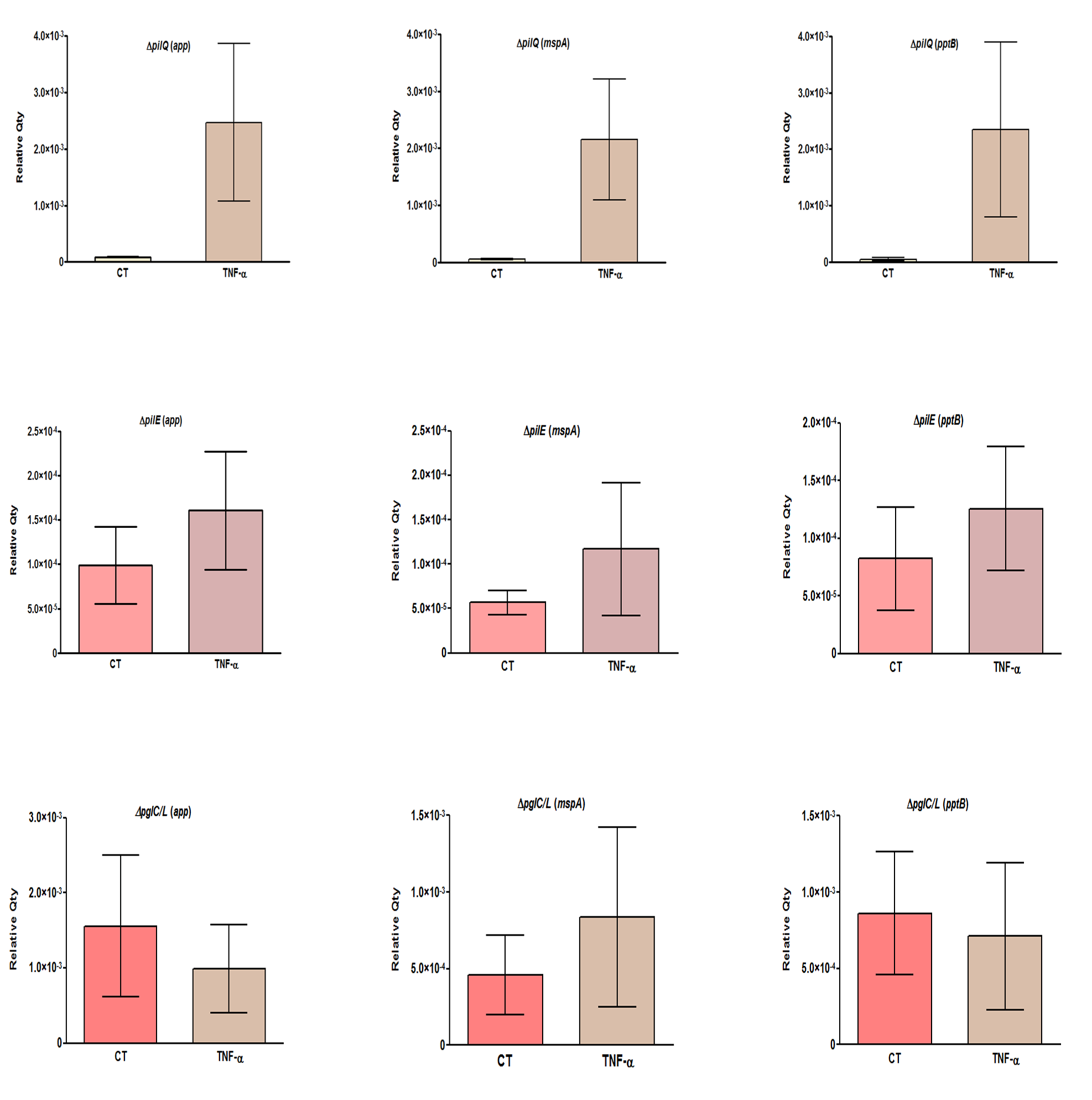


**S_8_-Figure.** The ∆*pilQ* mutant was cultured and induced with TNF-α (100 µg/ml) for 4h. The top panel depicts qPCR results, generated with primers designed to detect the open reading frame of *app* *mspA* *pptB* in each immunoprecipitate. Three independent experiments are shown for the IP study carried out in the presence and absence of human recombinant cytokines. Non-induced strains were considered as a negative control.

**Supplementary material 9:**

A study by Kallstrom and colleagues proposed that CD46 is one of the cellular receptors for Tfp [[50](#_ENREF_50)] and that after initial adhesion to host cell, polysaccharide capsule and Tfp expression are down-regulated [[51](#_ENREF_51)]. This is consistent with our deep sequencing results.

Disease progression was more efficient with piliated wild-type bacteria after intraperitoneal challenge of CD46 transgenic mice compared to the ∆*pilQ* and ∆*pglC/L* mutants, but not in case of ∆*pilE* mutant. In contrast to bacteraemia, which was significantly lower at 2 h post-challenge with ∆*pilE*, the disease progression was equally severe as with the WT strain (**S_9_**-**Table** ), indicating that PilQ protein expressed in ∆*pilE* is still competent to regulate the disease-causing virulent phenotype. The results of our TEM-∆*pilE* studies indicate that the quantities of ingested cytokines were not sufficient to be detected by TEM (**Fig. 3**) presumably because of less-efficient functional pili, but still a sufficient amount of cytokine uptake and consequent phenotypic modulation must be occurring to cause disease. ∆*pilQ* mutants expressing the PilE protein delayed disease progression, indicating again that defective pilus assembly and/or incorrect distribution of PilE protein on the surface plays

an important role in adaptation and competence for the establishment of disease. Interestingly, mice that were infected with the ∆*pglC/L* double mutant, expressing both PilQ and PilE proteins but lacking glycan moieties (**illustrated in S_10_-Fig.**), developed significantly less disease, indicating that glycans are essential for active uptake and that lack of glycans results in a reduced capacity to alter the

genes required to survive in a highly inflamed environment. These observations are entirely consistent with our TEM data (**Fig. 3**).

Inflammation is the innate immune response of the host to an infectious or non-infectious assault. The most proximal expression of such a response is the elaboration of the pro-inflammatory cytokines tumor necrosis factor alpha (TNF-α), interleukin-1β (IL-1β), interleukin-8 (IL-8), Interferon gamma (IFN-γ) and interleukin-6 (IL-6). When present at optimal concentrations, these biologically-active molecules

recruit both specific- and nonspecific-immune cells to the site of infection and activate them, thereby helping to eradicate the infection and restore homeostasis[[52](#_ENREF_52)].

**S_9_-Table. statistical analysis**

| Time(h) | Strains | Bacteraemia | IFN-γ | TNF-α | CXCL1 | IL-10 | IL-6 |
| --- | --- | --- | --- | --- | --- | --- | --- |
|  | MC58 vs ∆*pilE* | ***** | **ns** | **ns** | **ns** | **ns** | **ns** |
| 2 | MC58 vs ∆*pilQ* | **ns** | **ns** | **ns** | **ns** | **ns** | **ns** |
|  | MC58 vs ∆*pglL/C* | **ns** | **ns** | **ns** | ***** | **ns** | **ns** |
|  | MC58 vs ∆*pilE* | **ns** | **ns** | **ns** | **ns** | **ns** | **ns** |
| 6 | MC58 vs ∆*pilQ* | **ns** | **ns** | **ns** | **ns** | **ns** | **ns** |
|  | MC58 vs ∆*pglL/C* | **ns** | **ns** | **ns** | **ns** | **ns** | **ns** |
|  | MC58 vs ∆*pilE* | **ns** | **ns** | **ns** | **ns** | **ns** | **ns** |
| 24 | MC58 vs ∆*pilQ* | **ns** | **ns** | **ns** | **ns** | **ns** | **ns** |
|  | MC58 vs ∆*pglL/C* | **ns** | **ns** | **ns** | **ns** | **ns** | **ns** |
|  | MC58 vs ∆*pilE* | **ns** | **ns** | **ns** | **ns** | **ns** | **ns** |
| 48 | MC58 vs ∆*pilQ* | **ns** | **ns** | **ns** | **ns** | **ns** | **ns** |
|  | MC58 vs ∆*pglL/C* | **ns** | **ns** | **ns** | **ns** | **ns** | **ns** |

The CD46 transgenic mice were challenged intraperitoneally with *Neisseria* with 1.2x10^9^ CFU in 100 μl GC liquid medium. At the indicated time points, whole-blood samples were collected and bacteremia levels between MC58 wt and ***∆****pilQ*, ***∆****pilE,* and ***∆****pglL/C* mutant-infected animals were compared*.* Bacteria were detected in the blood 2 h after injection. At that time point, no significant differences in bacteria levels were observed between the tested strains. In contrast, 6 and 24 h after infection, the level of bacteria found in the blood was lower between the mice injected with ***∆****pilQ*, ***∆****pilE,* and ***∆****pglL/C* mutants, revealing their reduced ability to persist in the animals.

We also determined the blood concentration of the following inflammatory cytokines: TNF-α, IL-6, CXCL1 (KC), IL-10, and IFN-γ. As expected, we did not detect any cytokine production in the serum of uninfected mice (data not shown). High levels of IL-6, IL-10, and TNF-α were detected 2 h post challenge. IFN-γ production was slightly delayed and reached its maximum 6 h after bacterial injection. Whereas levels of IL-6, TNF-α, and IFN-γ decreased quickly over time, the concentration of KC remained rather stable 48 h after injection. Other cytokines, such as IL-1β and IL-12, were detected at very low levels in the plasma; thus, the corresponding data are not presented here.

We then compared the production of cytokines after infection with wt *Neisseria* or the ***∆****pilE,* ***∆****pilQ*, and ***∆****pglL/C* mutants. We found no significant differences in plasma cytokine concentrations between mice that were challenged with the WT strain MC58 and those challenged with the ***∆****pilE* mutant. In contrast, a reduction in cytokine production was observed in mice injected with ***∆****pilQ* (which exhibited reduced IFN-γ and CXCL1 levels) and ***∆****pilL/C* (which exhibited reduced IL-6, IFN-γ and CXCL1levels) mutants.

**Materials and Methods**

**Bacterial strains:**

*Neisseria meningitidis* strain MC58 was obtained from the ATCC (American Type Culture Collection). The mutant strains ∆*pglL*, ∆*pglC*, ∆*pglC*/*L* (double mutant), ∆*pilE*, ∆*pilQ*, ∆*lgtF,* and *siaD* were prepared in the laboratory, and all isolates were stored in glycerol at -80^o^C.

List of meningococcal strain MC58 mutants used in this study.

| Strains/plasmids | Description | Source or reference |
| --- | --- | --- |
| *N. meningitidis* | | |
| MC58 | wild-type serogroup B strain | [[42](#_ENREF_42)] |
| MC58Δ*PilQ* | *PilQ* deletion and replacement with omega cassette | [[53](#_ENREF_53)] |
| MC58Δ*PilE* | *PilE* deletion and replacement with a kanamycin cassette | In house |
| MC58Δ*PilC* | *PilC* deletion and replacement with an omega cassette | In house |
| MC58Δ*PglL* | *PglL*deletion and replacement with a kanamycin cassette | In house |
| MC58Δ*PglC* | *PglC* deletion and replacement with an omega cassette | In house |
| MC58Δ*PglL/C* | *PglL/C* deletion and replacement with an omega and kanamycin cassette | In house |

**Construction of MC58 ∆*pglL,* ∆*pglC* and a ∆*pglC/L* double mutant.**

A 3.3-kb DNA fragment consisting of the *pglC* gene and flanking DNA was amplified from *N. meningitidis* MC58 genomic DNA using primers pglC-F1 and pglC-R1 (Table 1). The amplified DNA was TA cloned into the pGEM-T Easy vector to generate pNJO98 (Table 2). This was then subject to inverse PCR using primers *pglC*-F2 and pglC-R2 (Table 1) resulting in the amplification of an amplicon in which the *pglC* coding sequence was deleted and a unique BamHI site had been introduced. The BamHI site was used to introduce a BamHI-digested spectinomycin/streptomycin cassette, originally from pHP45Ω (Table 2), in place of *pglC*. One of the resulting plasmids, pNJO100, was conﬁrmed by restriction digestion and sequencing and subsequently used to mutate the meningococcal strain MC58 by natural transformation and allelic exchange as previously described[[54](#_ENREF_54)].

A DNA fragment containing *pglL* was amplified from *Nm* MC58 DNA using the primers pglL-KO-F and pglL-KO-R, generating a 1944 bp product. This PCR product was cloned in pGEMT easy (promega) according manufactures instructions. The plasmid was linearized by an inverse PCR using the primers pglL-inv-F1 pglL-inv-R1. A blunt ended kanamycin cassette, digested from pJMK30 using SmaI, was inserted using T4 DNa ligase (promega). A clone with the kanamycin cassette in the same orientation as *pglL* was selected. The kanamycin cassette was introduced in MC58 WT and MC58Δ*pglC* the *pglC* using natural transformation by selection on BHI plates containing kanamycin 30 µg/ml, correct insertion in the genome was confirmed using primers located outside the recombination site; pglL-ors-F and pglL-ors-R.

**TABLE 1. List of primers used in this study**

| Primer | DNA sequence *^a^* | Restriction site |
| --- | --- | --- |
| pglC-F1 | TGCTGATGCAATATCTGCCGCTGTACG | Not present |
| pglC-R1 | GGTCATGACGTGTTCGAGCAGCGTGCG | Not present |
| pglC-R2 | CGC**GGATCC**CAAATTCGCGGCATTCGTTGCCCG | BamHI |
| pglC-F2 | CGC**GGATCC**GCTGTCGAGTTGGGCGACACCAGC | BamHI |
| pglL-KO-F | GCGCTTTCCGCAGTATTC | Not present |
| pglL-KO-R | TGTCTTGCATGGAGCTTTAC | Not present |
| pglL-inv-F1 | TTCGACGGCAGTTTCGTAG | Not present |
| pglL-inv-R1 | CAACGGCGGTTTCACAGAC | Not present |
| pglL-ors-F | TCCCGCAGAACAGATTTGC | Not present |
| pglL-ors-R | GCGTATTTCCCTACCGGTTTG | Not present |

*^a^* All primers were designed from the *N. meningitidis* MC58 genome sequence. Bold sequences identify restriction enzyme sites.

**TABLE 2. Bacterial strains and plasmids**

| Strain or plasmid | Description | Source or reference |
| --- | --- | --- |
| *E. coli* JM109 | *end*A1 *rec*A1 *gyr*A96 *thi hsd*R17 (r_K_^-^r_K_^-^) *rel*A1 *sup*E44 Δ(*lac*-*pro*AB) [F′ *tra*D36 *pro*AB *laq*I^q^ZΔM15] | Promega |
| *N. meningitidis* MC58 | wild-type serogroup B strain | [[42](#_ENREF_42)] |
| *N. meningitidis* MC58Δ*pglC* | *pglC* mutant derivative of MC58; streptomycin/spectinomycin resistant | This study |
| *N. meningitidis* MC58Δ*pglL* | *pglL* mutant derivative of MC58; kanamycin resistant | This study |
| *N. meningitidis* MC58Δ*pglL*  Δ*pglC* | *pglC and pglL* mutant derivative of MC58; streptomycin/spectinomycin and kanamycin resistant | This study |
| pGEM-T Easy | Cloning vector encoding resistance to ampicillin | Promega |
| pNJO98 | pGEM-T Easy plasmid containing *pglC* and flanking sequence | This study |
| pHP45Ω | Source of the streptomycin/spectinomycin cassette | [[55](#_ENREF_55)] |
| pNJO100 | pNJO98-derivative containing a streptomycin/spectinomycin cassette in place of *pglC* | This study |
| pJMK30 | Source of the kanamycin cassette | [[56](#_ENREF_56)] |
| pJS110 | pGEM-T Easy plasmid containing *pglL* and flanking sequence | This study |
| pJS111 | pJS110-derivative containing a kanamycin cassette inserted in the *pglL* gene | This study |

**Bacterial culture and cytokine induction:**

Successive passages of the wild type MC58, *ΔpilQ*, *ΔpilE, ΔpglC, ΔpglL*, and *ΔpglC, ΔpglL* (double mutant) strains were cultured on Columbia chocolate agar (Oxoid) and incubated overnight in a aerophilic cabinet (5% CO_2_) at 37^o^C. The following day, a loop-full of each strain was suspended in 10 ml of GIBCO® Dulbecco’s Modified Eagle Medium (DMEM) Media (Invitrogen) in 50 ml Falcon tubes. After mixing, the suspended colonies were allowed to grow for 4-5 hours to an OD_600_ of 0.1 – 0.9 in a shaking incubator at 37^o^C. The OD was measured at 600_nm_ using an IMPLEN OD_600_ Geneflow Spectrophotometer.

T-175 cm^2^ flasks were filled with 50 ml of DMEM medium, and inoculums (MC58, *ΔpilQ*, *ΔpilE* strains) from the 50 ml Falcon tubes were each added to one flask in volumes sufficient to achieve an OD_600_ of 0.1. The strains were then treated with purified TNF-α (20 ng/ml and 40 ng/ml) or IL-8 (40 ng/ml), while some cultures of the same series were untreated and used as a control. Flasks were incubated in aerophilic conditions at 37^o^C.

**Enzyme – linked immunosorbant assay (ELISA):**

Washes were performed at room temperature in PBS/T (0.1%) (Phosphate Buffered Saline/ Tween) using a Thermo Scientific Wellwash Versa. Incubations were performed on a platform agitator at room temperature, unless otherwise stated.

Purified, human recombinant IL-10, IL-12, IL-8 (aa 1-77), TNF-α (Super family, Member 2), and INF-γ (3 µg/ml) (Sino Biological Inc, Beijing, China) were coupled to 96-well plates (NUNC Immoblizer Amino) by adding 100 µl of cytokine solution (3 µg/ml) diluted in sodium carbonate buffer (pH=9) to each well. Negative control wells were filled with 100 µl BSA (Bovine Serum Albumin; 5 μg/ml) diluted in sodium carbonate buffer. The plates were incubated overnight at 4^o^C, and subsequently washed three times. Blocking was performed by adding 100 μl of 1% BSA in sodium carbonate buffer (100 µg/ml) to each well and incubating the plates for 1 h at room temperature.

The bacterial strains MC58, *ΔpilQ*, and *ΔpilE* were cultured on Columbia chocolate blood agar plates (Oxoid) for 40 h and washed 3 times in PBS/T. Isolates were then labeled with 10 μg/ml digoxigenin (Digoxigenin-3-O-methylcarbonyl-ε-aminocaproic acid-N-hydroxysuccinimide ester; Roche) in PBS for 2 h at room temperature. The labeled bacteria were washed three times with PBS/T and re-suspended in 1% BSA/PBS. The OD_600_ (optical density) was measured and adjusted to 0.02. A total of 100 µl of labeled bacteria were added to each well, and the plates were incubated overnight at 4^o^C.

The plates were then washed five times and incubated with a polyclonal anti-digoxigenin antibody Fab fragment that was conjugated with Alkaline phosphatase (1:5000; Roche) in 1% BSA/PBS (100 µl per well). The plates were then incubated for 1 h; after five additional washes, 100 µl of NBT solution (5 mg/ml; Roche) was added to each well. The absorbance at 405_nm_ was measured at after 1 h, and again after an additional 2 h. For inhibition assay, the bacterial cells was pre-incubated with a series of lectins for 2 h followed by several washes then the bacterial cells was added to coated plates with various cytokines. The following steps were similar to ELISA.

Each ELISA was run in triplicate, and a mean average absorbance was calculated for each condition. Specific binding was determined by subtracting the BSA binding value from the purified cytokines binding value for each bacterial strain.

List of meningococcal strain MC58 mutants used in this study.

| Abbreviations | Names | Specificity |
| --- | --- | --- |
| GSL-II | *Griffonia(Bandeiraea) simplicifolia* lectin II | **A galactosylated tri/tetra antennary glycans, GlcNAc** |
| DSL | *Datura Stramonium* lectin | **GlcNAc _β1-4_ ˃˃ GlcNAc** |
| ECL | *Erythrina cristagalli* lectin | **Gal_β1-4_GlcNAc-** |
| LEL | *Lycopersicon esculentum* (tomato) lectin | **GlcNAc- trimer/tetramer** |
| STL | *Solanum tuberosum* (potato) lectin | **GlcNAc oligomers** |
| VVA | *Vicia villosa* agglutinin | **α-linked terminal GalNAc, GalNAc_α1-3_Gal-** |
| Jacalin | *Artocarpus integrifolia* | **Gal_β1-3_GalNAc_α1_-Ser/Thr** |

**Expression and purification of recombinant protein, PilQ.**

Expression of the recombinant proteins that were cloned in pQE30 was performed using overnight cultures that were grown to exponential phase at 37°C as determined by OD at 600 nm of 0.6. Then, they were induced with IPTG for 3 h. Pre-induction and induced samples were solubilized with SDS samples buffer and run on 10% SDS-polyacrylamide gel together with negative control (*E. coli* strain JM109 containing pQE30 with no insert) prepared in the same manner. Band 80 kDa was observed in induced sample of cells harbouring plasmid encoding PilQ, but not in the non-induced samples or in the negative control.

This process was performed using Ni-NTA spin kit (Qiagen). Cell pellets were suspended in urea-containing buffer (Buffer B; 8 M urea, 10 mM Tris.Cl, 100 mM NaH2PO4, pH 8) and sonicated. Sonicated cells were centrifuged at 10,000 × *g* for 30 min to pellet the cellular debris. To the cleared lysate 20mM imidazole was added before loading the lysate onto the Ni-NTA column (Qiagen). Columns were then centrifuged at *ca.* 890 × *g* for 2 min. Columns were washed 4-6 times with Buffer C (8 M urea, 10 mM Tris.Cl, 100 mM NaH_2_PO_4_, pH 6.3) and proteins were finally eluted with Buffer E (8 M urea, 10 mM Tris.Cl, 100 mM NaH_2_PO_4_, pH 4.5).

**RNA isolation:**

RNA pellets were thawed on ice, and RNA extractions were performed. Enzymatic lysis of cells was achieved by 10 min incubation (at room temperature) with lysozyme (15 mg/ml), and QIAGEN Proteinase K in TE buffer. Total RNA from the bacterial lysate was subsequently purified using a QIAGEN RNeasy® Mini Kit according to the manufacturer’s instructions. Any residual genomic DNA was then digested by incubating the RNA samples with RNAase-free TURBO^TM^ DNase I (2 U/μl) (Ambion® Applied Biosystems) at 37°C for 30 min. RNA cleanup and concentration was then performed using a QIAGEN RNeasy® MinElute Cleanup Kit to obtain 13 μl of eluted RNA.

The purity of this RNA was quantified by measuring the optical density absorption ratio OD_260_: OD_280_ (with an ideal range of 1.8 to 2.0) using a Nanodrop 1000 Spectrophotometer*.* RNA quality was also verified using the Agilent RNA 6000 Pico Assay (Agilent 2100 Bioanalyser, and *2100 Expert Software*) to calculate an RNA Integrity Number (RIN) for each sample (ideal value ≥ 7).

**Quantitative polymerase chain reaction (qPCR)**

qPCR was performed using a AB7500 Real Time PCR System and Power SYBR^®^ Green PCR Master Mix (Applied Biosystems) according to the manufacturers’ instructions. Experiments were performed in triplicate.

Primers specific to *app*, *mspA* and *pptB* were designed in the laboratory using Primer Express software (Applied Biosystems) and were supplied by Eurofins MWG Operon. The primers were used at concentrations that were previously optimized in the laboratory (shown in Table 2).

| Target | Size of Product | Optimized  Concentration (µM) | Primer Sequence |
| --- | --- | --- | --- |
| *app* | 76 bp | 0.3 | Forward 5'-GTTTTTTACCAACAGGAGGCTCAT-3' |
|  |  | 0.9 | Reverse 5'-CACTTTTGCTTTTGGGCATCA-3' |
| *mspA* | 106 bp | 0.3 | Forward 5'-GGCGGATACTTGGGTGAAAA-3' |
|  |  | 0.3 | Reverse 5'-TTCTATGGCTGCTTCATTGGTTT-3' |
| *pptB* | 80 bp | 0.3 | Forward 5'-AAGGCACGGAAGTCATCATC-3' |
|  |  | 0.3 | Reverse 5'-CTGTTTGAGGTAGCGGAAGG-3' |

**Table 1.** Primer sequences and concentrations used to amplify corresponding genes extracted from chromatin immune-precipitation extracted DNA.

Reaction mixtures (25 *μl* total volume) were prepared as shown in Table 1. A non-template control mixture that contained all reagents except bacterial DNA was included to test for contamination or cross reaction. cDNA from one of the Δ*app*, Δ*mspA* samples was used as a negative control. Each sample of was run in triplicate (identical replicates) on PCR plates. The cycling program consisted of a 2 min hold at 50°C, a 10 min incubation at 95°C (to activate the Hotstart enzyme) and 40 cycles of denaturation at 95°C for 15 s, followed by annealing/extending at 60°C for 1 min.

| Reagent | *app* | *mspA* | *pptB* |
| --- | --- | --- | --- |
| cDNA | 2 ng | 2 ng | 2 ng |
| Power SYBR^®^ Green Mastermix | 12.5 μl | 12.5 μl | 0.75 μl |
| Forward Primer | 0.75 μl | 0.75 μl | 0.75 μl |
| Reverse Primer | 2.25 μl | 0.75 μl | 0.75 μl |
| RNAse-free Water | 7.5 μl | 9.0 μl | 9.0 μl |

**Table 2.** Composition of the reaction mixtures used for qPCR. The total volume of the reaction mixture was 25 μl, and the primers were used in the concentrations shown in Table 1.

Quantification of cDNA was performed via the standard curve method using Applied Biosystems Sequence Detection Software version 1.3.1. The standard curve was prepared using serial ten-fold dilutions of cDNA from an untreated MC58 control sample. The quantity of *app*, *mspA* and *pptB* cDNA in each sample was calculated relative to the standard curve.

This was achieved by calculating the cDNA quantities for *app, mspA* and *pptB*. A mean average was then calculated from the each of the triplicate samples. ANOVA tests, followed by Dunnett’s post-tests, were performed to assess the statistical significance of the change in cDNA levels for each condition. A probability level of p <0.05 was set.

**Immunogold labeling and electron microscopy:**

Wild-type and mutants (∆*pilQ*, ∆*pilE*, and ∆*pg1L/C*) *Neisseria meningitidis* were treated with either TNF-α or IL-8 or for 4-9 h, with untreated replicates included for each strain/treatment combination. Bacteria were then fixed in 3% paraformaldehyde and 0.1% glutaraldehyde in phosphate buffer for 2 h at room temperature and processed for transmission electron microscopy. The samples were subsequently washed, dehydrated and processed into Araldite resin blocks (TAAB laboratories), before being sectioned and mounted onto nickel grids. For the immunogold labeling, samples were washed (in a 1% BSA and 5% goat serum solution) and incubated overnight at 4^°^C with either anti-TNF-α or anti-IL-8 monoclonal antibodies at 2 µg/ml (Thermo Scientific), followed by labeling with goat anti-mouse IgG:10 nm gold (BB International) at 0.2 µg/ml for 4 h at 4^°^C. To prevent non-specific binding with both primary and secondary antibodies, BSA and goat serum were used at appropriate stages in the procedure. Imaging was performed on a FEI Technai 12 Biotwin transmission electron microscope at 100 kV.

# Quantification of gold particle in *Nm* samples induced either with IL-8 or TNF-α (immunogold-EM).

The tables below provide the number of grids, fields and experiments examined in this study.

| **IL8** | **No fields** | **Grids** | **No** |
| --- | --- | --- | --- |
| **MC58 induced** | 100 | 6 | 6 |
| **∆*pilQ* induced**  **treated** | 37 | 4 | 4 |
| **∆*pgIL/C* induced** | 20 | 3 | 3 |
| **AB controls** | 18 | 4 | 4 |
| **∆*pil*E induced** | 34 | 4 | 4 |
| **∆*pilT* induced** | 12 | 2 | 2 |

| **TNF-α** | **No fields** | **Grids** | **No** |
| --- | --- | --- | --- |
| **MC58 non-induced** | 16 | 3 | 2 |
| **MC58 induced** | 75 | 7 | 5 |
| **∆*pilQ* induced** | 50 | 7 | 5 |
| **∆*pilQ* non-induced** | 17 | 3 | 3 |
| **∆*pgIL/C* induced** | 34 | 4 | 4 |
| **AB controls** | 18 | 4 | 4 |
| **∆*pil*E induced** | 32 | 3 | 3 |
| **∆*pilE* untreated** | 8 | 1 | 1 |
| **∆*pilT* induced** | 16 | 2 | 2 |

# Negative staining

Bacteria were fixed in 3% glutaraldehyde and 1% paraformaldehyde in 0.1 M cacodylate buffer and subsequently washed 3 times in 0.1 M cacodylate buffer. A 20-µl bacterial suspension was placed on a formvar carbon-coated copper grid for 30 s before being drawn off with filter paper. A 20 µl drop of 2% neutral phosphtungstic acid was immediately added for 30 s and then drawn off with filter paper. The grids were allowed to dry before being imaged at 100 kV on an FEI Technai 12 Biotwin transmission electron microscope.

**Confocal microscopy**

For confocal microscopy, bacteria were grown in DMEM, collected and OD_600_ was adjusted with DMEM to 0.1-0.15. Bacteria were then induced with Atto680-labelled recombinant proteins (TNF-α or Galectin-3) at 100ng/ml and incubated at 37°C for 4 h. For inhibition of TNF-α uptake by bacteria, the equal amount of non-labeled TNF-α was added to the culture medium.

Cultures were harvested, washed twice with PBS (centrifugation 4000 × *rpm* for 10 min) and resuspended in 1 × PBS, at an OD_600_ of 1.0. 50 µl aliquots were then added to Knittel adhesive glass slides (SLS) and left at room temperature for 1 h in a humidified atmosphere before fixation with 4% paraformaldehyde for 10 min at RT. Slides were washed four times in 1 × PBS and then blocked with 1% BSA/PBS for 1 h. Membrane was visualized by: labeling surface-accessible PorA with anti-meningococcal sero-subtype P1.7 monoclonal antibody (1:10000; NIBSC code: 01/514), followed by 1× PBS wash and subsequent goat anti-mouse IgG-Alexa488 conjugate (1:400 diluted; Invitrogen) for 1 h. Following a final 1 × PBS wash, cover-slips were mounted with Prolong Gold anti-fade with DAPI (Invitrogen) and images acquired on a Zeiss LSM700 confocal microscope using a Plan-Apochromat 63x/1.40 Oil DIC M27 objective. Images are single sections (300_nm_) and data was collected from different fluorophores in separate channels. Images were processed using Image J and Adobe Photoshop.

**Chromatin IP**

The IP protocol used in this study was the same as described in Grainger et al., with slight modifications[[57](#_ENREF_57),[58](#_ENREF_58)]. Overnight cultures (start culture; OD_600_ 0.01) of *Nm* and the corresponding mutants in DMEM and a sub-culture in the same medium with and without induction with TNF-α for 4 h which was used for IP. The optical density of each of the bacterial cultures were quite similar (OD_600_ 0.33-0.34). The *in vivo* cross-linking of bacterial nucleoprotein was initiated via the addition of formaldehyde (at a final concentration of 1%) to cultures. After 20 min, cross-linking was quenched by adding glycine (at a final concentration of 0.5 M). The cells were then harvested from 30 ml culture samples by centrifugation, washed twice with Tris-buffered-saline (pH 7.5), resuspended in 1.5 ml of lysis buffer (10 mM Tris [pH 8.0], 20% sucrose, 50 mM NaCl, and 10 mM EDTA) and incubated at 37°C for 30 min. Following lysis, 4 ml of immunoprecipitation buffer (50 mM HEPES-KOH [pH 7.5], 150 mM NaCl, 1 mM EDTA, 1% Triton X-100, 0.1% sodium deoxycholate, 0.1% sodium dodecyl sulfate (SDS) and phenylmethylsulfonyl fluoride [at a final concentration of 1 mM]) were added. Cellular DNA was then sheared by sonication to an average size of 500 to 1,000 bp. Cell debris was removed by centrifugation (30 min at 4^°^C) and the supernatant was retained for use as the input sample for immunoprecipitation experiments.

A 300 µl aliquot of the input sample was used for each immunoprecipitation experiment. The sample was incubated overnight with mouse monoclonal anti-TNF-α antibody at a 1:5000 dilution (Thermo Fisher, catalogue no. MA1-22744) on a rotating wheel at 4^°^C.

An immunoprecipitation experiment using the same antibody in conjunction with non-induced bacterial cells was also performed as a negative control. Beads coated with goat anti mouse antibodies were then added to the samples for 2 h (5x10^6^ per sample) before being collected using a magnetic rake. This was followed by three washes with immunoprecipitation buffer and two washes with PBS-T. Immunoprecipitated complexes were then removed from the beads by treatment with 20 µl Proteinase K (20 mg/ml) (Sigma, UK) and incubated at 37°C overnight, followed by 2 h incubation at 65°C. Prior to analysis, DNA was purified from the immunoprecipitates using a PCR purification kit (QIAGEN). All ChIP assays were repeated at least 4-6 times, and the results were found to be reproducible within an error margin of 20-30%. The extracted DNA was analyzed by qPCR as described above.

**DNA Binding Studies**

Electrophoretic mobility shift assays (EMSA) were performed essentially as described[[59](#_ENREF_59)]. Briefly, synthetic oligonucleotides (NMB1985 adhesion and penetration protein, *app* promoter region. Overlapping fragments used here are: **Pro.1-2:** TTT CGG TTG TCC GTT TGT CGG TTG TTT TCA TTA TTT TTC CTT ATC TGA CGG GAT TCG GGT TTG TTT GGG AT, **Pro. 3-4:** CGG GAT TCG GGT TTG TTT GGG AGG GCG CGG CTT CCG CTT CCG GGC GGC GCG CGG GAT GTG CCT ATA TGT GCG GTT CGG CG, **Pro. 5-6:** TAT GTG CGG TTC GGC GTT CGG GCG GAT ATG AAG CAC GCC CTA GGA TTT GTC ATT AAT TTT TGC CTT GGT CTC GGC TTC TTC CA, **Internal region of *app* gene itself:** GTT TTT TAC CAA CAG GAG GCT CAT TTG GCG ACA GTG GCT CAC CAA TGT TTA TCT ATG ATG CCC AAA AGC AAA AGT G were amplified and incubated with 50,000 cpm (∼0.1 ng) of ^32^P-end-labeled oligonucleotide for 20–30 min at room temperature in 10 or 20 μl reaction volumes containing 12% glycerol, 12 mm HEPES-NaOH (pH 7.9), 60 mm KCl, 5 mm MgCl_2,_ 4 mm Tris-Cl (pH 7.9), 0.6 mm EDTA (pH 7.9), 0.6 mm dithiothreitol.

Protein-DNA complex was resolved in 6% polyacrylamide gels pre-electrophoresed for 30 min at room temperature in 0.25 × TBE buffer (22.5 mm Tris borate and 0.5 mm EDTA, pH 8.3). Gels were dried and exposed to radiographic film. Gel shifts were performed at least twice with using synthetic oligos at two different occasions. Similar results were obtained and a representative gel is shown in the figures.

**Deep sequencing**

Total RNA was enriched via two rounds of ribodepletion using a MICROBExpress kit (Ambion). Barcoded RNA-seq libraries were then constructed from each enriched RNA sample using a Total RNA-seq kit (Ambion) and sequenced using a SOLiD 4 genome analyzer (Applied Biosystems).

The SOLiD reads were mapped to the reference genome (*Neisseria meningitidis* MC58) using BioScope 1.3.1 software. The htseq-count script from the HTSeq Python package

(<http://www-huber.embl.de/users/anders/HTSeq/doc/count.html>) was used to count the number of reads that were mapped to each gene. The total number of reads per kilobase per million mapped reads (RPKM) were also calculated[[60](#_ENREF_60)]. Differential expression analysis between the samples was performed using the R package DEGseq[[61](#_ENREF_61)].

**Serum bactericidal assay**

Serum bactericidal assay (SBA) was performed as previously described [[62](#_ENREF_62)], following overnight incubation of wt MC58 and corresponding mutants on chocolate agar. Then bacteria were inoculated into Mueller-Hinton broth. The bacteria were grown for approximately for 4 h to early log phase, washed and resuspended in Dulbecco’s phosphate-buffered saline containing 9 mM CaCl2, 4.9 mM MgCl2 and 1% (w/v) bovine serum albumin. In this study, the SBA was performed collected at weeks 6 and all sera were heat inactivated for 50 min in 56 ^°^C. 10% (v/v) baby rabbit complement was used as an exogenous complement source [[17](#_ENREF_17),[63](#_ENREF_63),[64](#_ENREF_64)]. The buffer contained 25 µl of serially diluted sera in Dulbecco’s buffer, 12.5 µl of Dulbecco’s buffer containing 300 CFU of bacteria, and 12.5 µl of complement (20%, v/v). 10 µl aliquot of each well was spotted onto a chocolate agar plate and incubated overnight at 37 ^°^C in a 5% CO2 atmosphere. 96-well plates were incubated for 90 min at 37 ^°^C and then, a 10 µl aliquot was taken from each well and spotted onto a chocolate agar plates. The percentages of bacteria surviving were calculated by comparing the respective CFU at 90 min with that at time zero in negative control samples. Bactericidal titres were reported as the reciprocal of the highest dilution of test serum that yielded ≥50% bacterial killing compared to assay controls. All assays were performed in triplicate and repeated at three independent occasions.

**Reporter gene *lacZ* assay**

Promoter-*lacZ* fusions were constructed by inserting *N. meningitidis* promoter regions upstream of *lacZ*, creating promoter-lacZ translational fusions, using the *Bam*HI site upstream of *lacZ* in pLAS94 [[65](#_ENREF_65)]. For gene NMB0946 (preroxiredoxin, *prx*) the promoter was amplified with primers 5’-AAAAGGATCCAGCACCCAAATCCACA-3’ and 5’ AAAAGGATCCCCGGTACGATCTTGCAAA-3’. For gene NMB0750 (bacterioferritin co-migratory protein, *bcp*) the promoter was amplified with primers 5’ AAAAGGATCCACATCCATAGTCCTAC-3’ and 5’ AAAAGGATCCTGTGAGGAGAGAATC-3’. For gene NMB1998 (*mspA*) the promoter was amplified with 5’-CGCGGATCCCGCATGATGATTATCCGTGTA-3’ and 5’-CGCGGATCCAAC AACCGGAAAACGCAG-3’ by these sets of primers. Derivatives of pLES94 containing the promoter region were checked by sequencing and transformed into *N. meningitidis* MC58, and verified by PCR. β-galactosidase activity was assayed by the method of Miller [[66](#_ENREF_66)], after growth of *N. meningitidis* strains for 0-12 hours in DMEM medium in the presence or absence of cytokines IL-8 or TNF-α, both at a concentration of 100 ng/ml.

**Mouse model of infection**

The hCD46Ge transgenic mouse line (CD46^+/+^) was created using B6C3F1 hybrids. It harbors the complete human CD46 gene and expresses CD46 in a human-like pattern[[67](#_ENREF_67)]. Previous studies have shown that this mouse model can develop meningococcal disease [[68-70](#_ENREF_68)]. Serogroup B *N. meningitidis* MC58 and the mutant strains were grown for 18 h at 37°C in a 5% CO2 atmosphere on GC agar (a selective medium for the isolation of Neisseria species), (Difco) supplemented with Kellogg’s additive. The bacteria were then suspended in GC liquid, and each mouse was challenged intraperitoneally (i.p.) with 1.2x 10^9^ CFU in 100 μl of GC liquid medium. Experiments were performed with 6–8-week-old mice (n= 10 mice per group). In the control group, mice were challenged *i.p.* with 100 μl GC liquid. The health condition of all mice was closely monitored for 7 days. At the indicated time points, whole blood samples were collected from the tail vein for the measurement of cytokines, chemokines and bacteremial levels. The brain and spleen of each animal were collected at the end of the experiments and stored in 4% formaldehyde.

The mouse experiments described in the present study were performed at the animal facility of the Wenner-Grens Institute, Stockholm University. Animal care and experiments were conducted according to the institution’s guidelines for animal husbandry. All protocols were approved by the Swedish Ethical Committee on Animal Experiments (**Approval ID: N316/10**).

**Detection of meningococci in mouse blood**

A total of 5 μl of each blood sample was diluted in 245 μl of GC liquid and plated on GC agar plates following serial dilutions. The plates were then incubated overnight at 374^°^C in a 5% CO_2_ atmosphere, and the number of colony forming units (CFU) was counted the following day.

**Reference:**

1. Chi H, Flavell RA (2007) Immunology: sensing the enemy within. Nature 448: 423-424.

2. Le J, Vilcek J (1987) Tumor necrosis factor and interleukin 1: cytokines with multiple overlapping biological activities. Lab Invest 56: 234-248.

3. Kindler V, Sappino AP, Grau GE, Piguet PF, Vassalli P (1989) The inducing role of tumor necrosis factor in the development of bactericidal granulomas during BCG infection. Cell 56: 731-740.

4. Grau GE, Fajardo LF, Piguet PF, Allet B, Lambert PH, et al. (1987) Tumor necrosis factor (cachectin) as an essential mediator in murine cerebral malaria. Science 237: 1210-1212.

5. Tracey KJ, Fong Y, Hesse DG, Manogue KR, Lee AT, et al. (1987) Anti-cachectin/TNF monoclonal antibodies prevent septic shock during lethal bacteraemia. Nature 330: 662-664.

6. Lucas R, Magez S, De Leys R, Fransen L, Scheerlinck JP, et al. (1994) Mapping the lectin-like activity of tumor necrosis factor. Science 263: 814-817.

7. Estabrook MM, Jack DL, Klein NJ, Jarvis GA (2004) Mannose-binding lectin binds to two major outer membrane proteins, opacity protein and porin, of Neisseria meningitidis. J Immunol 172: 3784-3792.

8. Meduri GU, Kanangat S, Stefan J, Tolley E, Schaberg D (1999) Cytokines IL-1beta, IL-6, and TNF-alpha enhance in vitro growth of bacteria. Am J Respir Crit Care Med 160: 961-967.

9. Power PM, Roddam LF, Dieckelmann M, Srikhanta YN, Tan YC, et al. (2000) Genetic characterization of pilin glycosylation in Neisseria meningitidis. Microbiology 146 ( Pt 4): 967-979.

10. Stimson E, Virji M, Makepeace K, Dell A, Morris HR, et al. (1995) Meningococcal pilin: a glycoprotein substituted with digalactosyl 2,4-diacetamido-2,4,6-trideoxyhexose. Mol Microbiol 17: 1201-1214.

11. Chamot-Rooke J, Rousseau B, Lanternier F, Mikaty G, Mairey E, et al. (2007) Alternative Neisseria spp. type IV pilin glycosylation with a glyceramido acetamido trideoxyhexose residue. Proc Natl Acad Sci U S A 104: 14783-14788.

12. Marceau M, Forest K, Beretti JL, Tainer J, Nassif X (1998) Consequences of the loss of O-linked glycosylation of meningococcal type IV pilin on piliation and pilus-mediated adhesion. Mol Microbiol 27: 705-715.

13. Nothaft H, Szymanski CM (2010) Protein glycosylation in bacteria: sweeter than ever. Nat Rev Microbiol 8: 765-778.

14. Spillmann D, Witt D, Lindahl U (1998) Defining the interleukin-8-binding domain of heparan sulfate. J Biol Chem 273: 15487-15493.

15. Kuschert GS, Hoogewerf AJ, Proudfoot AE, Chung CW, Cooke RM, et al. (1998) Identification of a glycosaminoglycan binding surface on human interleukin-8. Biochemistry 37: 11193-11201.

16. Hebert CA, Vitangcol RV, Baker JB (1991) Scanning mutagenesis of interleukin-8 identifies a cluster of residues required for receptor binding. J Biol Chem 266: 18989-18994.

17. Clark-Lewis I, Schumacher C, Baggiolini M, Moser B (1991) Structure-activity relationships of interleukin-8 determined using chemically synthesized analogs. Critical role of NH2-terminal residues and evidence for uncoupling of neutrophil chemotaxis, exocytosis, and receptor binding activities. J Biol Chem 266: 23128-23134.

18. Frevert CW, Kinsella MG, Vathanaprida C, Goodman RB, Baskin DG, et al. (2003) Binding of interleukin-8 to heparan sulfate and chondroitin sulfate in lung tissue. Am J Respir Cell Mol Biol 28: 464-472.

19. Geoffroy MC, Floquet S, Metais A, Nassif X, Pelicic V (2003) Large-scale analysis of the meningococcus genome by gene disruption: resistance to complement-mediated lysis. Genome Res 13: 391-398.

20. Shevchenko A, Wilm M, Vorm O, Mann M (1996) Mass spectrometric sequencing of proteins silver-stained polyacrylamide gels. Anal Chem 68: 850-858.

21. Wosten MM (1998) Eubacterial sigma-factors. FEMS Microbiol Rev 22: 127-150.

22. Ren J, Sainsbury S, Combs SE, Capper RG, Jordan PW, et al. (2007) The structure and transcriptional analysis of a global regulator from Neisseria meningitidis. J Biol Chem 282: 14655-14664.

23. Perez-Rueda E, Collado-Vides J (2000) The repertoire of DNA-binding transcriptional regulators in Escherichia coli K-12. Nucleic Acids Res 28: 1838-1847.

24. Sigmund CD, Morgan EA (1988) Nus A protein affects transcriptional pausing and termination in vitro by binding to different sites on the transcription complex. Biochemistry 27: 5622-5627.

25. Ma JC, Newman AJ, Hayward RS (1981) Internal promoters of the rpoBC operon of Escherichia coli. Mol Gen Genet 184: 548-550.

26. Andre G, Even S, Putzer H, Burguiere P, Croux C, et al. (2008) S-box and T-box riboswitches and antisense RNA control a sulfur metabolic operon of Clostridium acetobutylicum. Nucleic Acids Res 36: 5955-5969.

27. Winkler W, Nahvi A, Breaker RR (2002) Thiamine derivatives bind messenger RNAs directly to regulate bacterial gene expression. Nature 419: 952-956.

28. Echenique-Rivera H, Muzzi A, Del Tordello E, Seib KL, Francois P, et al. (2011) Transcriptome Analysis of Neisseria meningitidis in Human Whole Blood and Mutagenesis Studies Identify Virulence Factors Involved in Blood Survival. PLoS Pathog 7: e1002027.

29. Whitchurch CB, Hobbs M, Livingston SP, Krishnapillai V, Mattick JS (1991) Characterisation of a Pseudomonas aeruginosa twitching motility gene and evidence for a specialised protein export system widespread in eubacteria. Gene 101: 33-44.

30. Wolfgang M, Lauer P, Park HS, Brossay L, Hebert J, et al. (1998) PilT mutations lead to simultaneous defects in competence for natural transformation and twitching motility in piliated Neisseria gonorrhoeae. Mol Microbiol 29: 321-330.

31. Tonjum T, Caugant DA, Dunham SA, Koomey M (1998) Structure and function of repetitive sequence elements associated with a highly polymorphic domain of the Neisseria meningitidis PilQ protein. Mol Microbiol 29: 111-124.

32. Pujol C, Eugene E, Marceau M, Nassif X (1999) The meningococcal PilT protein is required for induction of intimate attachment to epithelial cells following pilus-mediated adhesion. Proc Natl Acad Sci U S A 96: 4017-4022.

33. Parkhill J, Achtman M, James KD, Bentley SD, Churcher C, et al. (2000) Complete DNA sequence of a serogroup A strain of Neisseria meningitidis Z2491. Nature 404: 502-506.

34. Serruto D, Adu-Bobie J, Scarselli M, Veggi D, Pizza M, et al. (2003) Neisseria meningitidis App, a new adhesin with autocatalytic serine protease activity. Mol Microbiol 48: 323-334.

35. Turner DP, Marietou AG, Johnston L, Ho KK, Rogers AJ, et al. (2006) Characterization of MspA, an immunogenic autotransporter protein that mediates adhesion to epithelial and endothelial cells in Neisseria meningitidis. Infection & Immunity 74: 2957-2964.

36. Dietrich G, Kurz S, Hubner C, Aepinus C, Theiss S, et al. (2003) Transcriptome analysis of Neisseria meningitidis during infection. J Bacteriol 185: 155-164.

37. Virji M (2009) Pathogenic neisseriae: surface modulation, pathogenesis and infection control. Nat Rev Microbiol 7: 274-286.

38. van Ulsen P, Tommassen J (2006) Protein secretion and secreted proteins in pathogenic Neisseriaceae. FEMS Microbiol Rev 30: 292-319.

39. Jordan PW, Saunders NJ (2009) Host iron binding proteins acting as niche indicators for Neisseria meningitidis. PLoS One 4: e5198.

40. Yao H, Jepkorir G, Lovell S, Nama PV, Weeratunga S, et al. (2011) Two distinct ferritin-like molecules in Pseudomonas aeruginosa: the product of the bfrA gene is a bacterial ferritin (FtnA) and not a bacterioferritin (Bfr). Biochemistry 50: 5236-5248.

41. Schryvers AB, Stojiljkovic I (1999) Iron acquisition systems in the pathogenic Neisseria. Mol Microbiol 32: 1117-1123.

42. Tettelin H, Saunders NJ, Heidelberg J, Jeffries AC, Nelson KE, et al. (2000) Complete genome sequence of Neisseria meningitidis serogroup B strain MC58. Science 287: 1809-1815.

43. Spinosa MR, Progida C, Tala A, Cogli L, Alifano P, et al. (2007) The Neisseria meningitidis capsule is important for intracellular survival in human cells. Infect Immun 75: 3594-3603.

44. Stevanin TM, Moir JW, Read RC (2005) Nitric oxide detoxification systems enhance survival of Neisseria meningitidis in human macrophages and in nasopharyngeal mucosa. Infect Immun 73: 3322-3329.

45. Stefanelli P, Colotti G, Neri A, Salucci ML, Miccoli R, et al. (2008) Molecular characterization of nitrite reductase gene (aniA) and gene product in Neisseria meningitidis isolates: is aniA essential for meningococcal survival? IUBMB Life 60: 629-636.

46. Ku SC, Schulz BL, Power PM, Jennings MP (2009) The pilin O-glycosylation pathway of pathogenic Neisseria is a general system that glycosylates AniA, an outer membrane nitrite reductase. Biochem Biophys Res Commun 378: 84-89.

47. Shafer WM, Veal WL, Lee EH, Zarantonelli L, Balthazar JT, et al. (2001) Genetic organization and regulation of antimicrobial efflux systems possessed by Neisseria gonorrhoeae and Neisseria meningitidis. J Mol Microbiol Biotechnol 3: 219-224.

48. Fantappie L, Metruccio MM, Seib KL, Oriente F, Cartocci E, et al. (2009) The RNA chaperone Hfq is involved in stress response and virulence in Neisseria meningitidis and is a pleiotropic regulator of protein expression. Infect Immun 77: 1842-1853.

49. Born TL, Blanchard JS (1999) Structure/function studies on enzymes in the diaminopimelate pathway of bacterial cell wall biosynthesis. Curr Opin Chem Biol 3: 607-613.

50. Kallstrom H, Liszewski MK, Atkinson JP, Jonsson AB (1997) Membrane cofactor protein (MCP or CD46) is a cellular pilus receptor for pathogenic Neisseria. Mol Microbiol 25: 639-647.

51. Deghmane AE, Giorgini D, Larribe M, Alonso JM, Taha MK (2002) Down-regulation of pili and capsule of Neisseria meningitidis upon contact with epithelial cells is mediated by CrgA regulatory protein. Mol Microbiol 43: 1555-1564.

52. Meduri GU, Estes RJ (1995) The pathogenesis of ventilator-associated pneumonia: II. The lower respiratory tract. Intensive Care Med 21: 452-461.

53. Orihuela CJ, Mahdavi J, Thornton J, Mann B, Wooldridge KG, et al. (2009) Laminin receptor initiates bacterial contact with the blood brain barrier in experimental meningitis models. J Clin Invest 119: 1638-1646.

54. Hadi HA, Wooldridge KG, Robinson K, Ala'Aldeen DA (2001) Identification and characterization of App: an immunogenic autotransporter protein of *Neisseria meningitidis*. Mol Microbiol 41: 611-623.

55. Prentki P, Krisch HM (1984) In vitro insertional mutagenesis with a selectable DNA fragment. Gene 29: 303-313.

56. van Vliet AH, Wooldridge KG, Ketley JM (1998) Iron-responsive gene regulation in a campylobacter jejuni fur mutant. J Bacteriol 180: 5291-5298.

57. Grainger DC, Overton TW, Reppas N, Wade JT, Tamai E, et al. (2004) Genomic studies with Escherichia coli MelR protein: applications of chromatin immunoprecipitation and microarrays. J Bacteriol 186: 6938-6943.

58. Waldminghaus T, Skarstad K (2010) ChIP on Chip: surprising results are often artifacts. BMC Genomics 11: 414.

59. Roebuck KA, Rahman A, Lakshminarayanan V, Janakidevi K, Malik AB (1995) H2O2 and tumor necrosis factor-alpha activate intercellular adhesion molecule 1 (ICAM-1) gene transcription through distinct cis-regulatory elements within the ICAM-1 promoter. J Biol Chem 270: 18966-18974.

60. Mortazavi A, Williams BA, McCue K, Schaeffer L, Wold B (2008) Mapping and quantifying mammalian transcriptomes by RNA-Seq. Nat Methods 5: 621-628.

61. Wang L, Feng Z, Wang X, Zhang X (2010) DEGseq: an R package for identifying differentially expressed genes from RNA-seq data. Bioinformatics 26: 136-138.

62. Brown DR, Helaine S, Carbonnelle E, Pelicic V (2010) Systematic functional analysis reveals that a set of seven genes is involved in fine-tuning of the multiple functions mediated by type IV pili in Neisseria meningitidis. Infection & Immunity 78: 3053-3063.

63. George AJ (1994) Surface-bound cytokines--a possible effector-mechanism in bacterial immunity. Immunol Today 15: 88-89.

64. Schmidt MA, Riley LW, Benz I (2003) Sweet new world: glycoproteins in bacterial pathogens. Trends Microbiol 11: 554-561.

65. Zhang Q, Meitzler JC, Huang S, Morishita T (2000) Sequence polymorphism, predicted secondary structures, and surface-exposed conformational epitopes of Campylobacter major outer membrane protein. Infect Immun 68: 5679-5689.

66. Paget MS, Helmann JD (2003) The sigma70 family of sigma factors. Genome Biol 4: 203.

67. Mrkic B, Pavlovic J, Rulicke T, Volpe P, Buchholz CJ, et al. (1998) Measles virus spread and pathogenesis in genetically modified mice. J Virol 72: 7420-7427.

68. Johansson L, Rytkonen A, Bergman P, Albiger B, Kallstrom H, et al. (2003) CD46 in meningococcal disease. Science 301: 373-375.

69. Johansson L, Rytkonen A, Wan H, Bergman P, Plant L, et al. (2005) Human-like immune responses in CD46 transgenic mice. J Immunol 175: 433-440.

70. Sjolinder H, Jonsson AB (2007) Imaging of disease dynamics during meningococcal sepsis. PLoS One 2: e241.
