# Supplementary figures and images for: Pro-inflammatory cytokines can act as intracellular modulators of commensal bacterial virulence
Source: Open Biol. 2013 Oct;3(10):130048. doi: 10.1098/rsob.130048 (PMC3814720; doi:10.1098/rsob.130048)

## Slide 1
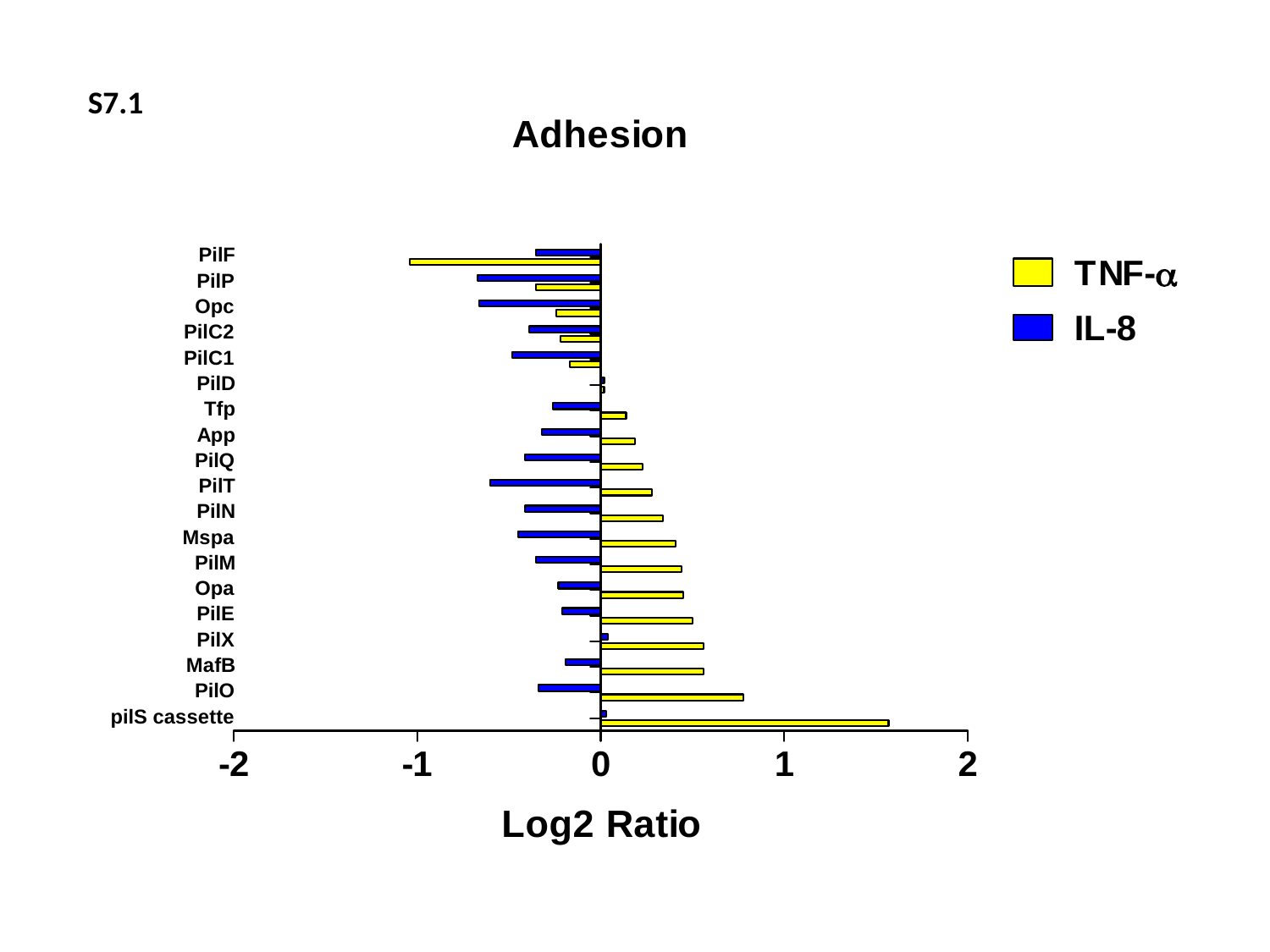

S7.1

## Slide 2
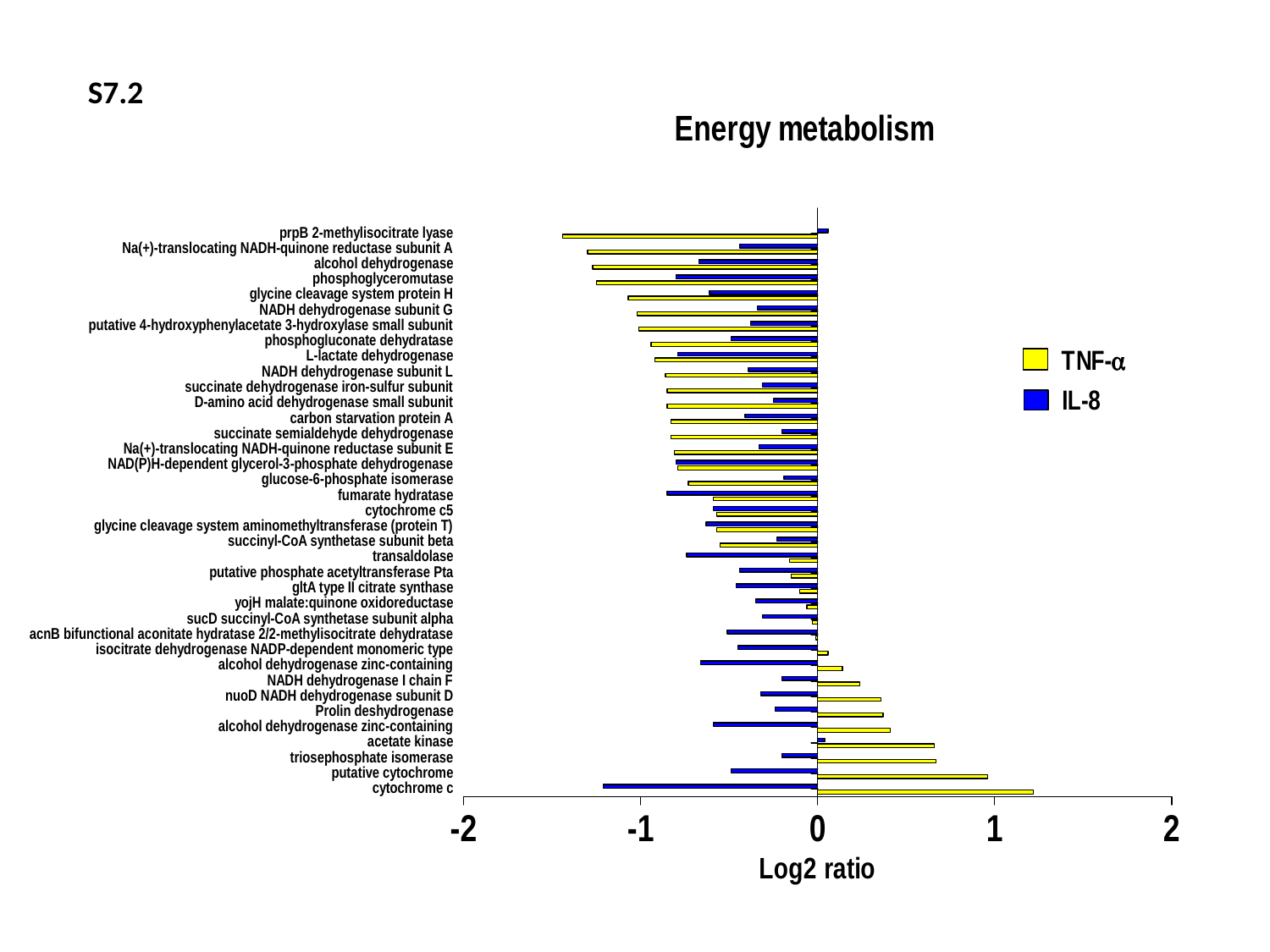

S7.2

## Slide 3
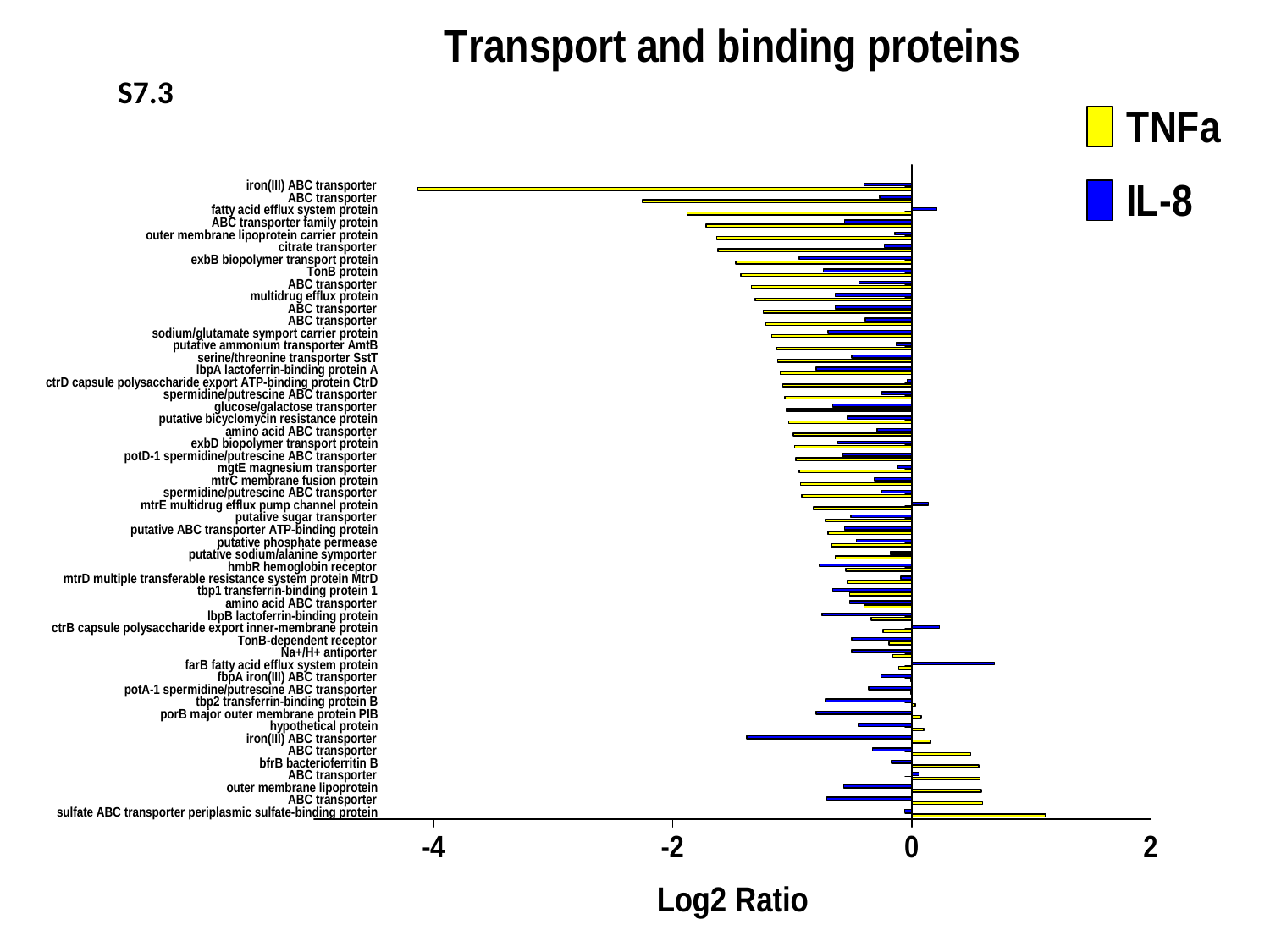

S7.3

## Slide 4
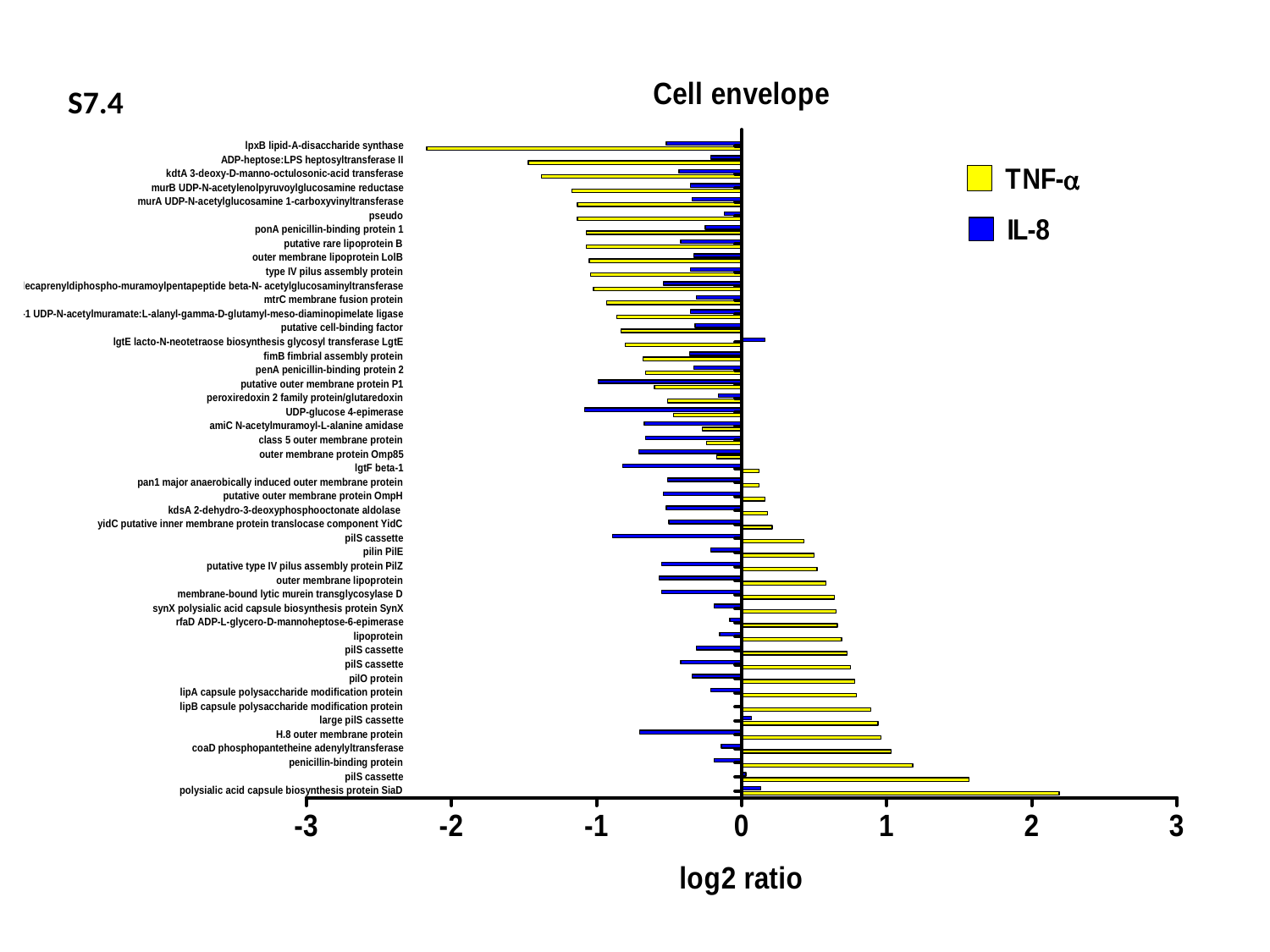

S7.4

## Slide 5
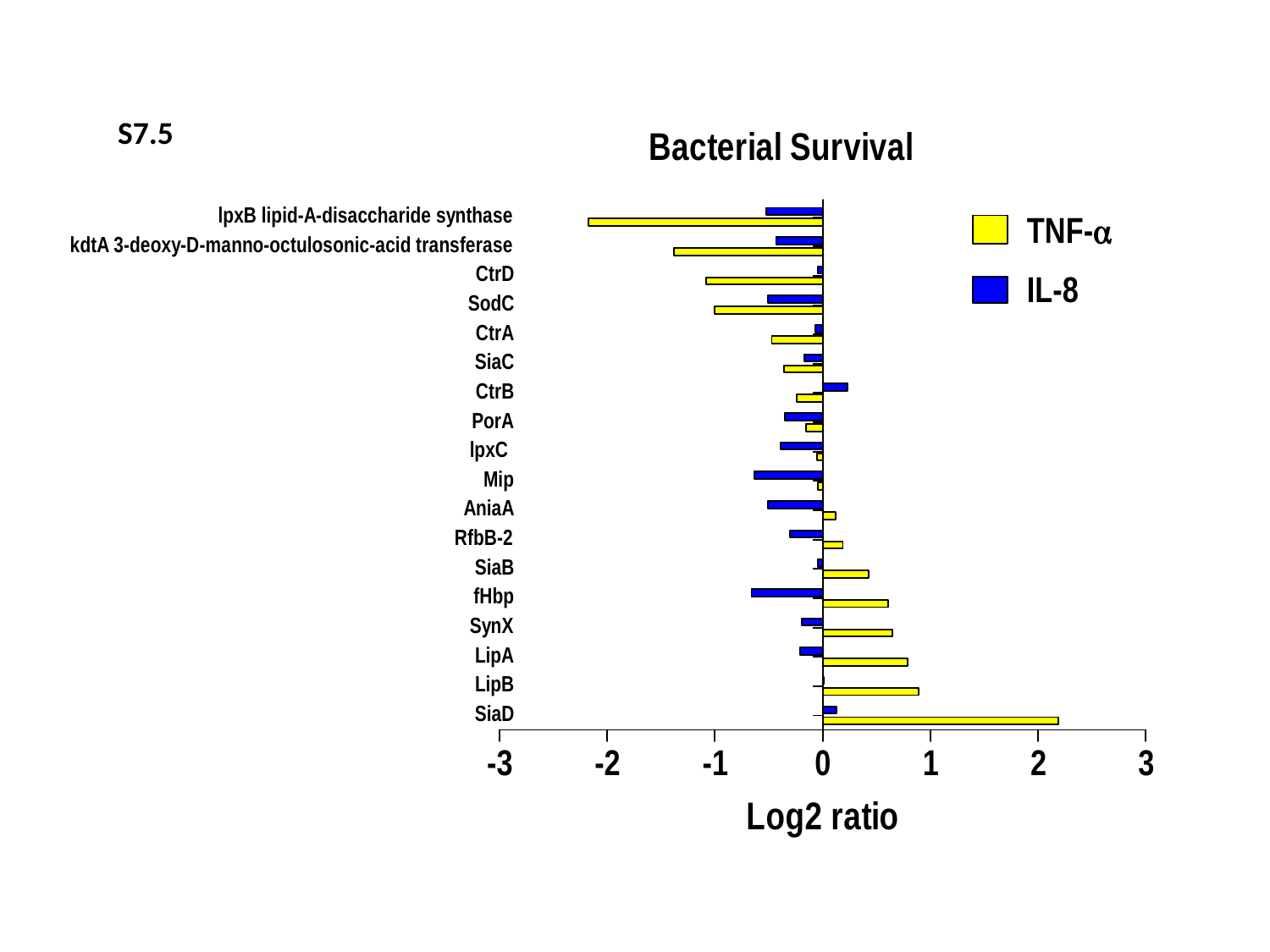

S7.5

## Slide 6
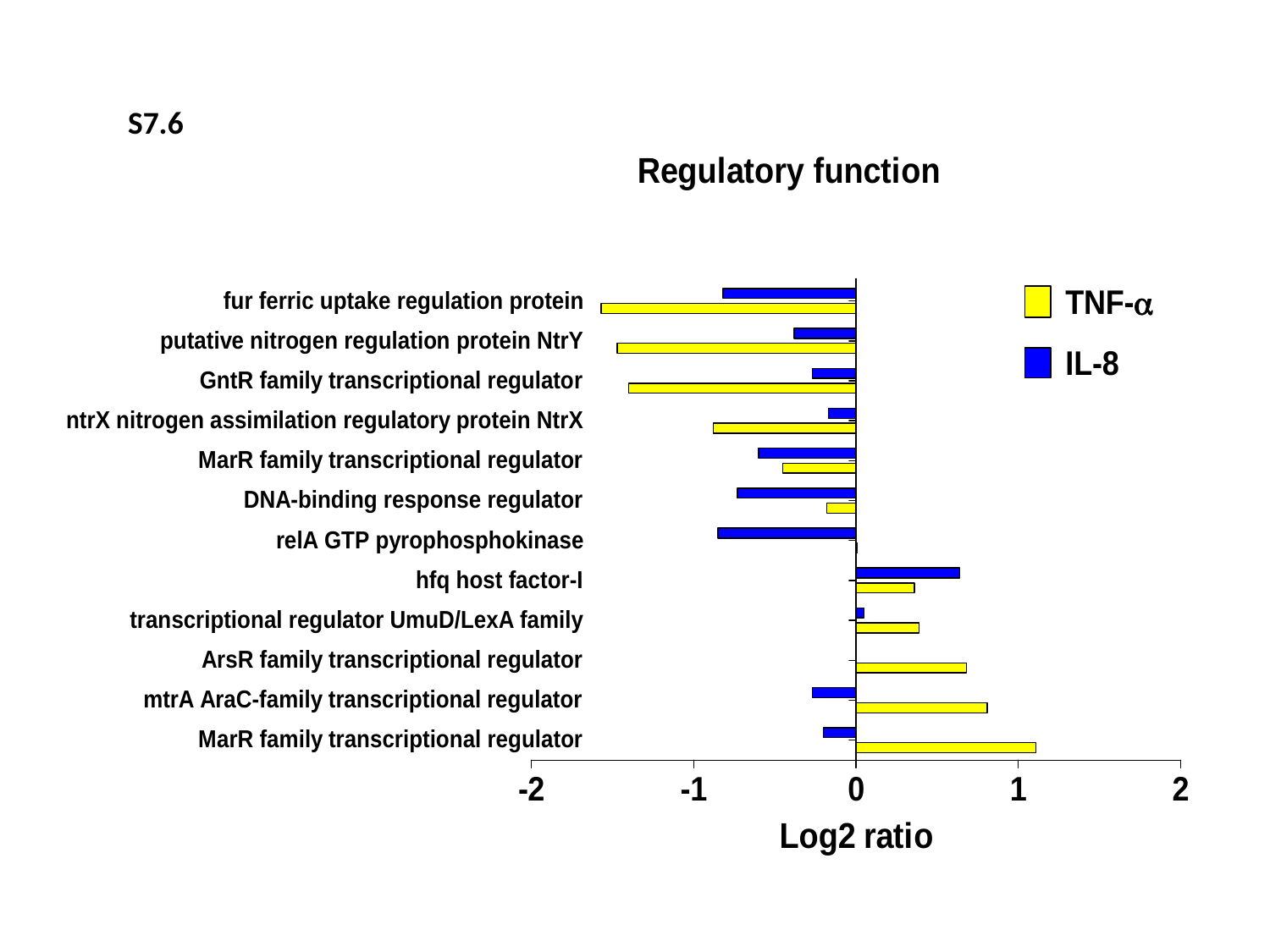

S7.6

## Slide 7
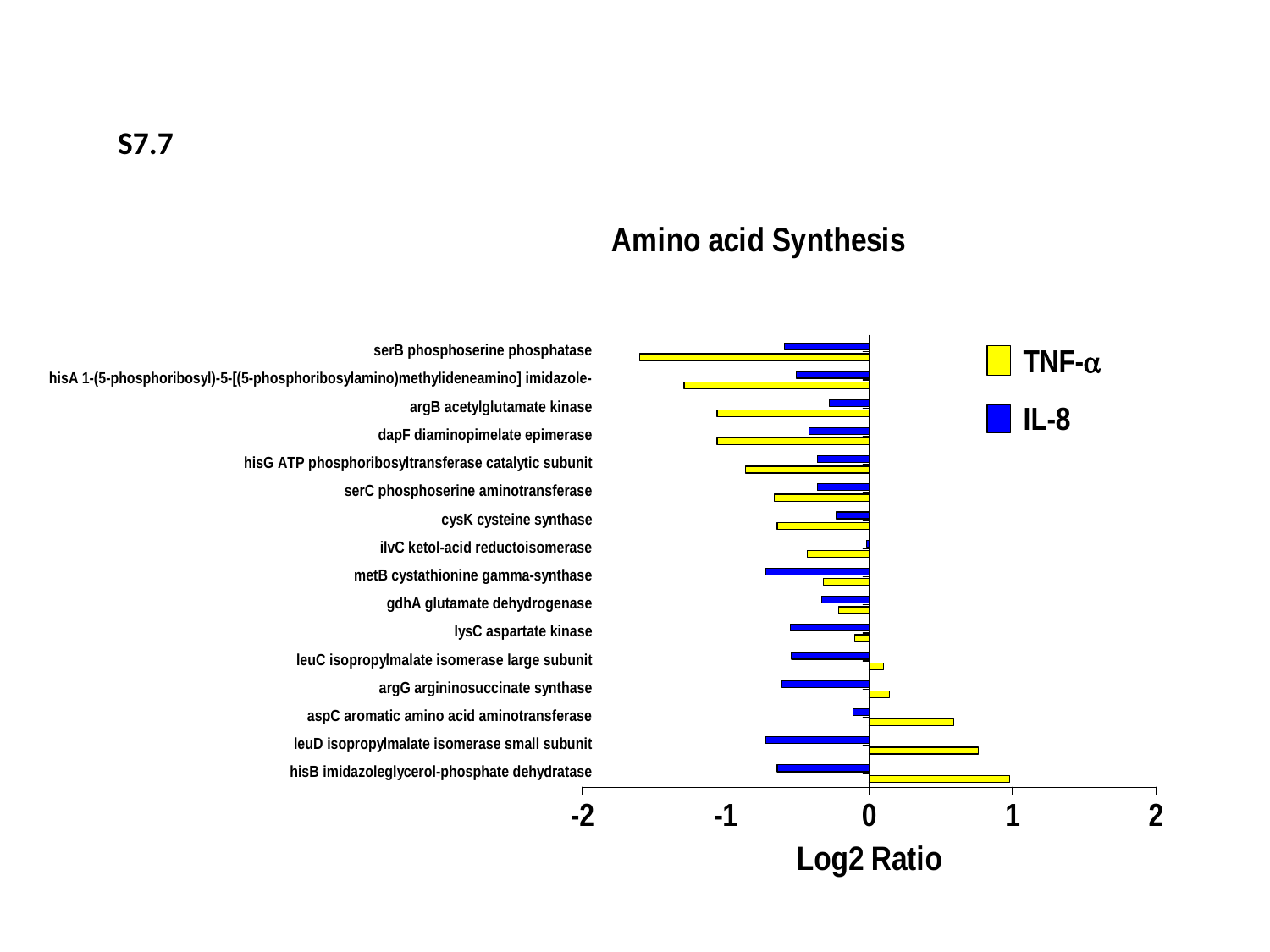

S7.7

Supplement: Deep Sequencing Figures [file rsob130048supp3.pptx]
